# Cognitive Training for Attention-Deficit/Hyperactivity Disorder: Meta-Analysis of Clinical and Neuropsychological Outcomes From Randomized Controlled Trials

**DOI:** 10.1016/j.jaac.2014.12.010

**Published:** 2015-03

**Authors:** Samuele Cortese, Maite Ferrin, Daniel Brandeis, Jan Buitelaar, David Daley, Ralf W. Dittmann, Martin Holtmann, Paramala Santosh, Jim Stevenson, Argyris Stringaris, Alessandro Zuddas, Edmund J.S. Sonuga-Barke, European ADHD Guidelines Group (EAGG)

**Affiliations:** a Developmental Brain-Behaviour Laboratory, University of Southampton, UK; b School of Medicine, University of Nottingham, UK; c New York University Child Study Center, New York; d Ghent University, Belgium; e Aarhus University, Denmark; f School of Medicine, University of Nottingham, UK and the Centre for ADHD and Neurodevelopmental Disorders Across the Lifespan, Institute of Mental Health, University of Nottingham, UK; g King's College London, Institute of Psychiatry, UK; h Centro de Salud Mental de Estella, Navarra, Spain; i Huntercombe Hospital Maidenhead, UK; j Central Institute of Mental Health, Medical Faculty Mannheim, Heidelberg University, Germany; k University of Zurich, Switzerland; l Donders Institute for Brain, Cognition and Behaviour, Radboud University Medical Centre, Nijmegen, The Netherlands; m Karakter Child and Adolescent Psychiatry University Centre, Nijmegen; n LWL-University Hospital for Child and Adolescent Psychiatry, Ruhr University, Bochum, Germany; o Unit of Child Neuropsychiatry, University of Cagliari, Cagliari, Italy

**Keywords:** ADHD, nonpharmacological, working memory, executive functions, evidence-based psychiatry

## Abstract

**Objective:**

The authors performed meta-analyses of randomized controlled trials to examine the effects of cognitive training on attention-deficit/hyperactivity disorder (ADHD) symptoms, neuropsychological deficits, and academic skills in children/adolescents with ADHD.

**Method:**

The authors searched Pubmed, Ovid, Web of Science, ERIC, and CINAHAL databases through May 18, 2014. Data were aggregated using random-effects models. Studies were evaluated with the Cochrane risk of bias tool.

**Results:**

Sixteen of 695 nonduplicate records were analyzed (759 children with ADHD). When all types of training were considered together, there were significant effects on total ADHD (standardized mean difference [SMD] = 0.37, 95% CI = 0.09–0.66) and inattentive symptoms (SMD = 0.47, 95% CI = 0.14–0.80) for reports by raters most proximal to the treatment setting (i.e., typically unblinded). These figures decreased substantially when the outcomes were provided by probably blinded raters (ADHD total: SMD = 0.20, 95% CI = 0.01–0.40; inattention: SMD = 0.32, 95% CI = −0.01 to 0.66). Effects on hyperactivity/impulsivity symptoms were not significant. There were significant effects on laboratory tests of working memory (verbal: SMD = 0.52, 95% CI = 0.24–0.80; visual: SMD = 0.47, 95% CI = 0.23–0.70) and parent ratings of executive function (SMD = 0.35, 95% CI = 0.08–0.61). Effects on academic performance were not statistically significant. There were no effects of working memory training, specifically on ADHD symptoms. Interventions targeting multiple neuropsychological deficits had large effects on ADHD symptoms rated by most proximal assessors (SMD = 0.79, 95% CI = 0.46–1.12).

**Conclusion:**

Despite improving working memory performance, cognitive training had limited effects on ADHD symptoms according to assessments based on blinded measures. Approaches targeting multiple neuropsychological processes may optimize the transfer of effects from cognitive deficits to clinical symptoms.

Attention-deficit/hyperactivity disorder (ADHD) is a childhood-onset condition characterized by pervasive patterns of inattention and/or impulsivity-hyperactivity that often persist into later life.^1^ Combinations of pharmacological and psychological approaches are recommended for its treatment.^2^ Although medication is efficacious in randomized controlled trials (RCT) in the short/medium-term and is indicated as the first-line treatment (at least for severe cases^2^), it has a number of potential limitations—each affecting some patients. These include the following: partial response or nonresponse^3^; possible adverse effects^4^; uncertainty about long-term costs and benefits^5^; poor adherence^6^; and negative medication-related attitudes from patients, parents, or clinicians.^7^ Psychological treatments such as behavioral parent training are also widely used. However, a recent meta-analysis^8^ found no effects on ADHD symptoms when only ratings by assessors blind to treatment allocation were considered.

In recent years, cognitive training has been investigated as a potential ADHD treatment.^9^ Building on evidence of brain plasticity from rehabilitation science and contemporary developmental neuroscience, cognitive training is premised on the notion that key brain networks implicated in ADHD can be strengthened, and the cognitive processes they subserve improved, through controlled exposures to information processing tasks.^10^ Thus, it is argued that cognitive training can reduce ADHD symptoms and improve functioning by targeting neuropsychological deficits thought to mediate ADHD pathophysiology. In keeping with the complex nature of ADHD neuropsychology,^11^ cognitive training approaches have targeted a range of deficits (e.g., attentional control, working memory, inhibitory control). Currently, such training is typically delivered via computers using adaptive procedures, whereby training task difficulty is automatically increased across sessions to continually challenge the patient at the boundaries of his or her competence. This has been shown in neuroimaging studies to be necessary for sustaining neuronal changes.^12,13^

The efficacy of cognitive training for ADHD was addressed in a meta-analysis of nonpharmacological treatments for ADHD by Sonuga-Barke *et al.*^14^ on behalf of the European ADHD Guidelines Group (EAGG). This meta-analysis focused solely on RCTs. Importantly, it addressed the issue of blinding by comparing outcomes rated by individuals most proximal to the therapeutic setting (often unblinded and invested in the patient and/or intervention) and those provided by reporters judged to be probably blinded. Effects of cognitive training on ADHD symptoms calculated using unblinded ratings were highly significant (standardized mean difference [SMD] = 0.64, 95% CI = 0.33–0.95). These effects dropped substantially (SMD = 0.24) and became statistically nonsignificant (95% CI = −0.24 to 0.72) when probably blinded measures were used. However, these results should be considered as preliminary because only 6 RCTs were included. The authors concluded that more evidence was required, especially from trials in which assessments were effectively blinded, before cognitive training could be supported as an ADHD treatment. A second meta-analysis by Rapport *et al.*,^9^ published more recently and exploring a wider range of outcomes, found similar effects. However, compared to Sonuga-Barke *et al.*,^14^ this more recent meta-analysis included only 2 additional peer-reviewed RCTs with outcomes related to ADHD core symptoms. Moreover, to increase statistical power, Rapport *et al.*^9^ also included non-RCTs and pooled across design types, making effect size estimates of the effects of cognitive training on ADHD core symptoms and related neuropsychological impairment difficult to interpret.

A significant number of new RCTs of cognitive training for ADHD, not available for inclusion in these previous 2 meta-analyses,^9,14^ have been published in the past 2 years, reflecting the current interest in cognitive training in this field. The greater number of trials now available allows a much more definitive estimate of the effects of cognitive training to be made. In the present article, we update the first EAGG cognitive training meta-analysis to include these new trials, and we extend its focus to cover effects on neuropsychological processes and academic functioning, which were not addressed in the previous EAGG meta-analysis.^14^ The focus on neuropsychological processes is important for 2 reasons. First, neuropsychological deficits are postulated to mediate the pathways between originating causes and disorder onset: improvements in neuropsychological functioning may therefore be a prerequisite for ADHD symptom reduction.^15^ Second, they are associated with functional impairments in their own right, independent of their association with ADHD symptomatology, especially in social and academic contexts.^16^ A broad range of training approaches have been used with ADHD populations. In the meta-analysis by Sonuga-Barke *et al.*,^14^ given the small number of studies available, trials with different techniques had to be pooled to generate an effect size estimate. However, given the increased number of trials now available, our aim was to explore training-type specific effects through the use of subanalyses where sufficient numbers of trials existed.

## Method

The EAGG protocol for nonpharmacological interventions for ADHD was registered on the International Prospective Register of Systematic Reviews PROSPERO (http://www.crd.york.ac.uk/PROSPERO, protocol number: CRD42011001393). The same protocol was followed here.

### Inclusion and Exclusion Criteria

Only RCTs including interventions aimed to directly train a cognitive function were retained. As reported by the Cochrane group,^17^ to ensure high levels of methodological adequacy and to avoid the inevitable bias caused by dependence on investigators agreeing to provide data from unpublished studies, only published studies were included. Trials were included if participants had an ADHD diagnosis (any subtype) or met accepted cut-offs on validated ADHD rating scales and were between 3 and 18 years of age. Trials involving children with ADHD comorbid with rare disorders (e.g., fragile X syndrome) only were excluded. Control conditions allowed were “treatment as usual,” “wait list,” “active/placebo/sham” (i.e., involving other forms of computer-based activity or alternative training regimen). Trials were not excluded if patients received medication as part of normal treatment. In an extension of the EAGG protocol,^14^ trials could be included in this updated meta-analysis despite not reporting an ADHD outcome if they reported neuropsychological and/or academic outcomes.

### Search Strategy

Sonuga-Barke *et al.*^14^ included studies up to April 3, 2012. Here, using the same search strategy, our final search date was May 18, 2014. Supplement 1, available online, reports details about the search strategy and syntax for each database. Parallel searches were conducted separately by the first 2 authors.

### Outcome Measures

For consistency with previous EAGG meta-analyses^8^ and to provide a robust estimate of effects, outcome domains were analyzed only if 5 or more RCTs were available. The outcomes analyzed were: ADHD symptoms (total ADHD as well as inattention and hyperactivity/impulsivity symptoms), parent ratings of executive functioning (e.g., Behavior Rating Inventory of Executive Function [BRIEF]), standardized measures of reading and arithmetic ability, and laboratory-based measures of verbal and visual working memory, inhibition, and attention. For neuropsychological outcomes, only scores from tasks different from those used for training were included in the analysis.

### Study Selection

Articles’ titles and abstracts were screened independently by the first 2 authors. Final inclusion was based on the full text. Trials were blindly double coded for eligibility by the first 2 authors (S.C., M.F). Disagreement was resolved by the senior author for 3 trials.

### Risk of Bias Assessment

Two authors independently assessed trial risk of bias using 5 domains of the Cochrane Collaborations tool^17^: namely, selection bias, performance bias, detection bias, attrition bias, and other bias. If there was disagreement between the 2 raters, the final rating was established through consensus with the involvement of the senior author. This occurred for 4 trials.

### Data Extraction and Statistical Analysis

Trial information was entered into RevMan 5.0.^18^ Data extraction was independently performed and cross-checked by the first 2 authors. SMD was calculated as mean pre- to posttreatment change in the intervention group minus the mean pre- to posttreatment change in the control group divided by the pooled pretest standard deviation with a bias adjustment.^19^ SMDs for each trial were combined using the inverse variance method. Given the inherent heterogeneity of studies, random effects models were used. The I^2^ statistic was calculated a posteriori to estimate between-trial SMD heterogeneity. For the most proximal analysis, parent ratings, if available, were used for home-based interventions, and teacher ratings were used for school-based interventions, except when it could be inferred from the manuscript’s text that teachers were less blinded than parents for home-based interventions and parents less blinded than teachers for school-based interventions (2 trials^20,21^). Probably blinded assessments were those made by an individual judged likely to be unaware of treatment allocation. In trials in which more than 1 such measure was available, the best-blinded measure was chosen. For home-delivered interventions, teachers’ ratings were usually judged to be blinded, whereas for school-based interventions, parents were judged to be blinded except where this could be inferred not to be the case from the text^20,21^ or from e-mail exchange with the authors. As per protocol, where direct observations were available, we selected these over rating-scale scores. This decision was based on the judgement that direct observations are likely, in general, to be better blinded than parent- or teacher-rated outcomes, even when the latter are made in a setting other than the therapeutic setting. Where multiple measures were available for a single outcome (as was sometimes the case for laboratory tasks), the measure most frequently reported across included trials and/or that which was judged to tap the core of the construct was selected. Sensitivity analyses were conducted including only trials meeting the following criteria: use of active/sham control; use of working memory training; use of training targeting more than 1 neuropsychological domain (termed here “multiple process training”); and use of no/low medication (i.e., with <30% of participants receiving medications). We also performed an additional sensitivity analysis excluding the study by Gray *et al.*,^22^ in which all participants had a diagnosis of ADHD plus coexisting intellectual disability. Publication bias was assessed with funnel plots and Egger’s tests. Finally, we also conducted a meta-regression analysis, using the *metareg* command in STATA,^23^ to assess the relationship between age and SMD for most proximal and probably blinded assessments of ADHD core symptoms. This analysis was conducted to establish whether the efficacy of cognitive training varied across age, a finding that could be of clinical significance.

## Results

**Figure 1:**
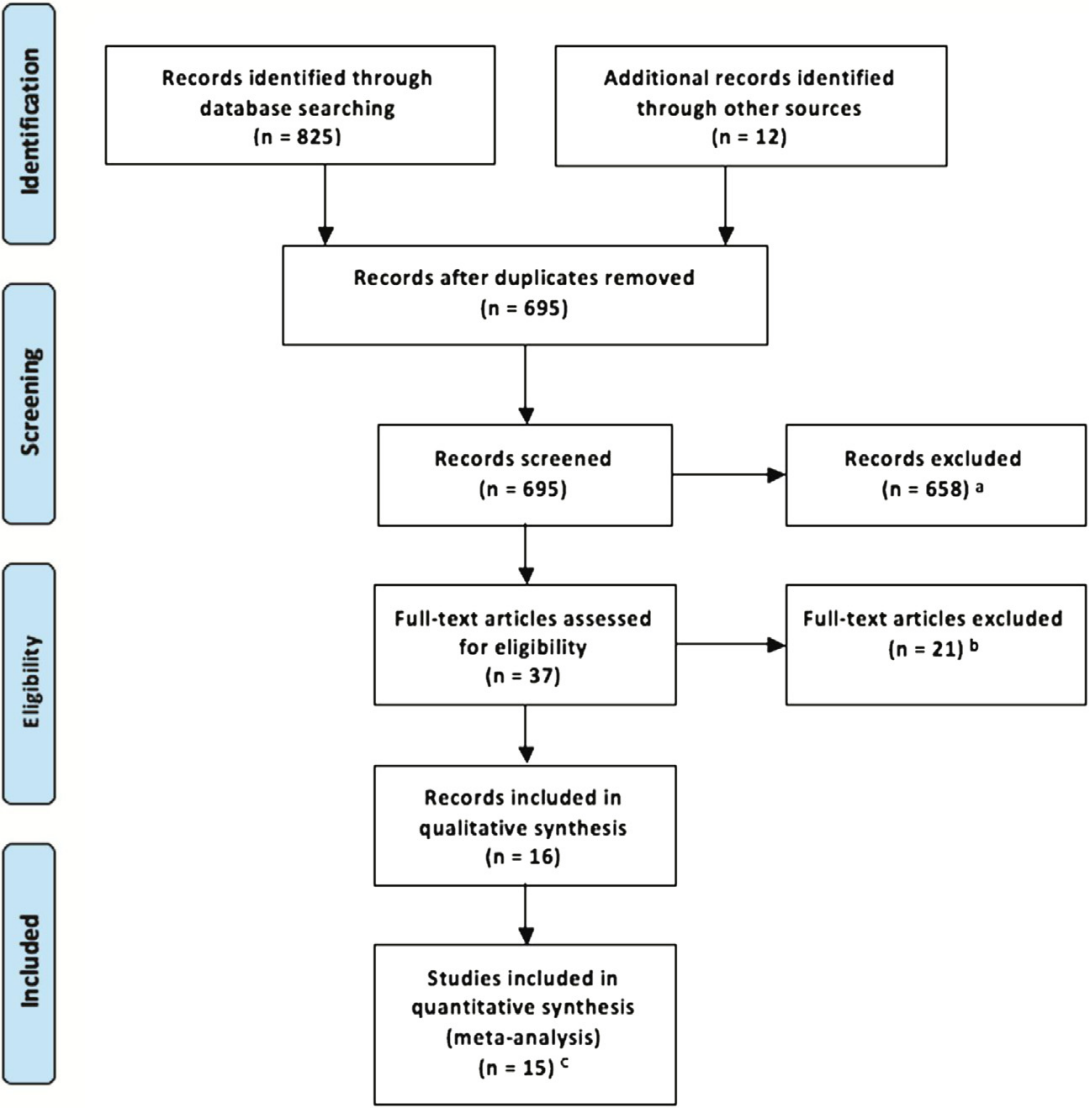
Preferred Reporting Items in Systematic Reviews and Meta-Analyses (PRISMA) flow diagram of selection of studies (last search updated May 18, 2014). Note: ^a^A total of 259 studies were not on attention-deficit/hyperactivity disorder (ADHD); 342 were not on cognitive training; 7 were not randomized controlled trials (RCTs); 47 were reviews; 3 were studies in adults; and 1 was a study protocol. ^b^Reasons for exclusion of each study are reported in Table S2, available online. ^c^Egeland *et al*.^24^ and Hovik *et al*.^25^ refer to the same study.

Fifteen trials (reported in 16 papers) met entry criteria (see Table S1, available online). Studies not included in the meta-analysis are listed (with reasons for their exclusion) in Table S2 (available online). Figure 1 reports the trial selection flowchart. Table 1 gives information about retained trials. Results of all analyses are summarized in Table 2. Six trials were on working memory training, 4 on attention training, 2 combined attention and working memory training, 2 inhibition and working memory training, and 1 trial provided a general executive function training covering working memory, inhibition, and cognitive flexibility. All training schedules had an “adaptive” component, that is, task difficulty was increased across sessions to track performance improvement. Eight trials had an active control condition. Six trials were implemented at home, 5 at school, 2 at either school or home, 1 trial in the clinic, and 1 at the welfare service/children center, home or laboratory. Five trials had no/low medication levels. Figure S1 (available online) depicts the graphic output for the risk of bias assessment. Risk of bias was generally low or unclear. No trials were scored as “high risk” with regard to “random sequence generation,” “allocation concealment,” and “incomplete outcome data,” and only 3 and 4 trials scored high for “blinding of participants/personnel” and “blinding of outcome assessment,” respectively (the rating of each study is available upon request).

### ADHD Symptoms

ADHD symptoms (total score or inattention or hyperactivity/impulsivity separately) were an outcome in up to 14 trials. Probably blinded measures were available in up to 11 trials (Table 2).

**Figure 2:**
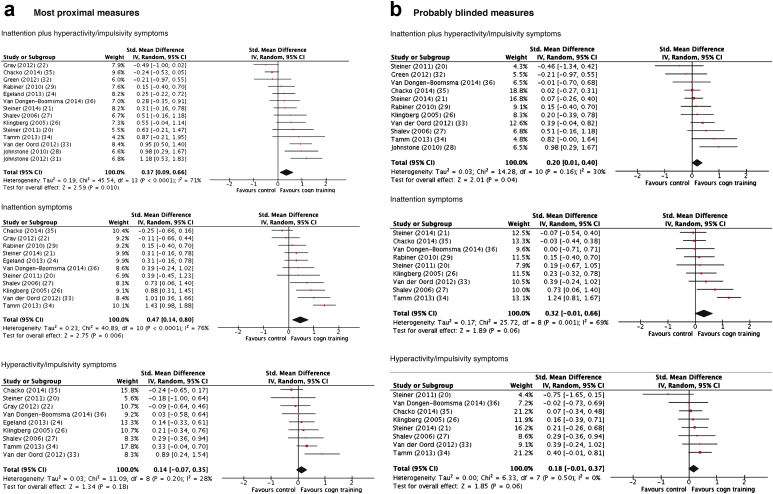
Forest plots for meta-analysis of effects on attention-deficit/hyperactivity disorder (ADHD) core symptoms assessed by the most proximal and probably blinded raters. Note: Cogn = cognitive; std = standard.

When most proximal assessments were the outcome, there was a moderate but significant effect on total ADHD and inattention symptoms but no effect on hyperactivity/impulsivity (Figure 2; SMD and CI data for all outcomes are presented in Table 2). In sensitivity analyses (Figures S2 and S3, available online), considering only trials with an active control, the effects were no longer statistically significant for any ADHD core symptoms outcomes. There was no effect of working memory training when implemented on its own (Figures S2 and S3, available online). In contrast, multi-process training approaches (i.e., approaches targeting more than 1 neuropsychological domain) gave a large effect size for total ADHD symptoms (Figure S2, available online). Between-study heterogeneity of effect sizes was high and significant for total ADHD and inattention symptoms.

When analyses were restricted to probably blinded measures (Figure 2), in general, effect sizes were reduced with small and statistically marginal effects for all ADHD outcomes. In a sensitivity analysis (Figure S4, available online), effect sizes dropped further to nonsignificant levels when only trials with an active control arm were included. There were insufficient studies (n < 5) for an analysis of probably blinded measures in multi-component training trials, as well as for a number of other sensitivity analyses.

When analysis was restricted to no/low medication trials, effects on total ADHD symptoms were not significant for either most proximal or, when available, probably blinded assessments in any ADHD core symptom–related outcome (Table 2).

### Neuropsychological Outcomes

**Figure 3:**
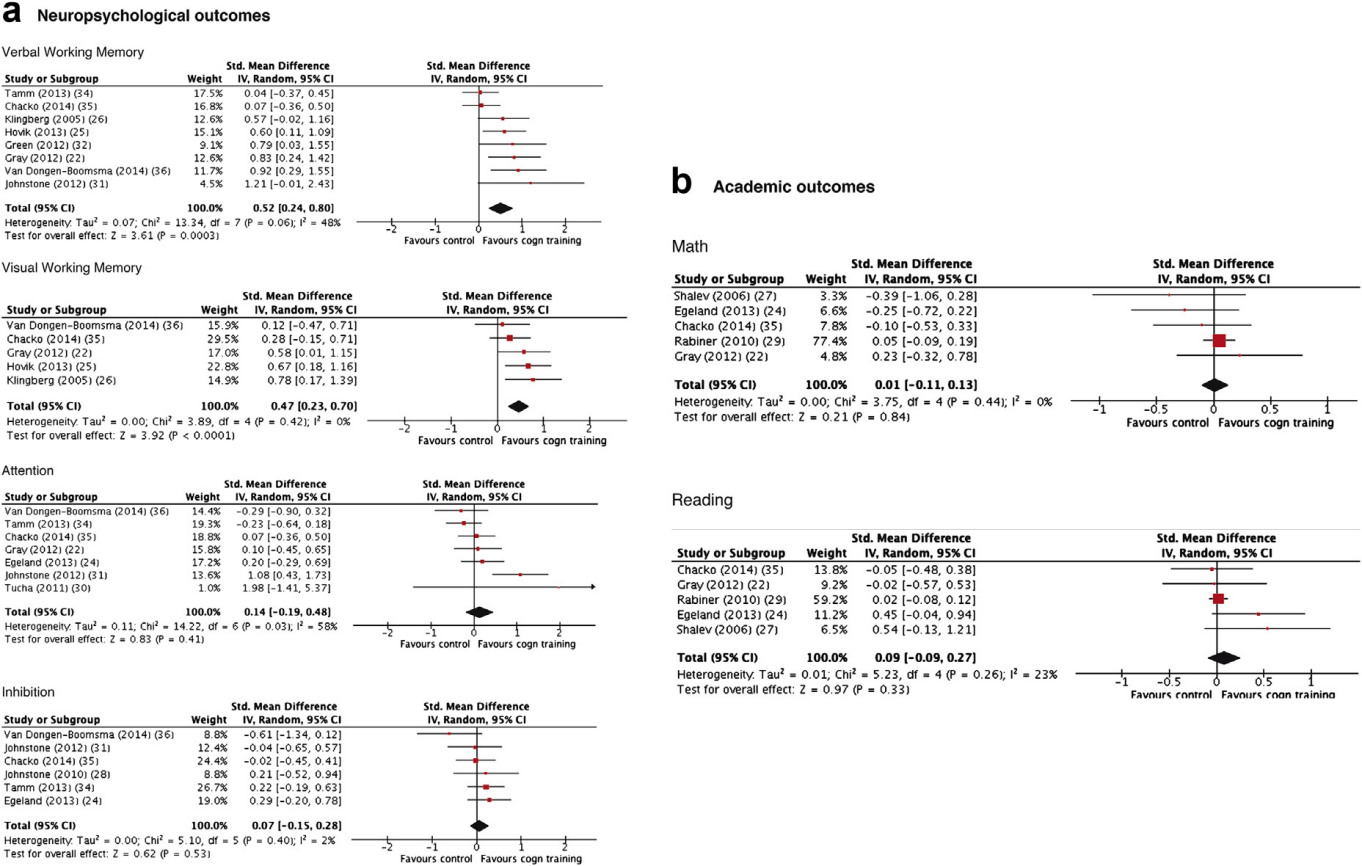
Forest plots for meta-analysis of effects on neuropsychological and academic outcomes. Note: Cogn = cognitive; std = standard.

Eight trials included laboratory measures of verbal, and 5 trials visual working memory (Table 2). There was a large and significant effect of cognitive training on both components (Figure 3), which was maintained in sensitivity analyses considering trials with active controls only or working memory training trials only (Figure S5, available online; sensitivity analyses were not performed for visual working memory because of an insufficient number of trials). The number of trials using multi-component training and no/low medication trials was insufficient to perform sensitivity analyses. There were no significant effects of training on laboratory tests of inhibition (6 trials) or attention (7 trials) (Figure 3). Six trials included most proximal ratings of executive functioning using the BRIEF rating scale (Figure S6, available online). These demonstrated a small-to-moderate, significant SMD. There was an insufficient number of trials with ratings of executive functioning to perform planned sensitivity analyses.

### Academic Ability

Five trials included standardized measures of reading and 5 of arithmetic. There were no significant effects in either domain (Figure 3). There was an insufficient number of trials to perform planned sensitivity analyses.

### Publication Bias

Funnel plots and results of Egger’s test are reported in Supplement 2 (available online). For both meta-analyses of ADHD symptoms scored by most proximal and probably blinded raters, the test failed to reach the *p* < 0.05 level, suggesting no significant publication bias.

### Meta-Regression Analysis

For most proximal or probably blinded assessments of ADHD core symptoms, there was no significant effect of age on SMD (see Supplement 3, available online).

### Sensitivity Analysis Excluding the Study by Gray *et al.*^22^

The main results considering most proximal assessment of ADHD core symptoms were substantially unchanged, as reported in Figure S7 (available online). As this study was not included in “probably blinded” analyses, no sensitivity analysis was conducted considering probably blinded assessment.

## Discussion

There are 2 perspectives on cognitive training in ADHD. From 1 perspective, cognitive training is a front-line ADHD treatment: this is based on the hypothesis that because causal pathways to disorder are mediated by neuropsychological deficits, strengthening deficient neuropsychological functions should reduce ADHD symptoms and associated impairment. From the second perspective, it is perceived as an adjunctive treatment that reduces impairment associated with neuropsychological deficits commonly seen in children with ADHD, independent of any effects on core ADHD symptoms itself. The current meta-analysis, including an additional 10 RCTs compared to the previous study by Sonuga-Barke *et al.*,^14^ provided little support for cognitive training as a front-line ADHD treatment. There were statistically significant effects on ADHD symptoms when considering raters most proximal to treatment delivery, especially for symptoms of inattention. However, these effects were reduced substantially when analyses were limited to trials with an active control arm or where assessors were probably blind to treatment allocation. The evidence was somewhat stronger for the benefits of cognitive training as an adjunctive treatment aimed at reducing neuropsychological impairment. There were large and highly significant improvements on objective tests of both visual and verbal working memory, although there were no effects on inhibition or inattention. Furthermore, the effects of cognitive training on working memory did not extend to the academic outcomes explored.

The substantial drop in SMDs between most proximal and probably blinded analyses for ADHD symptoms is similar to the pattern seen in previous meta-analyses of nonpharmacological treatments using the EAGG protocol (e.g., behavioral intervention;^8^ neurofeedback^14^). This is probably caused by the inflation of effect size estimates that inevitably occurs when one relies on raters who are both likely to be aware of treatment allocation and heavily invested in the delivery and outcome of treatment. It is also possible that probably blinded and most proximal assessments differed in some way that reduced the sensitivity of the former to treatment-related change. However, the same measurement approaches were used for each (some parent, some teacher, and some direct observation measures). Another possibility is that most proximal assessments accurately captured treatment effects established in the therapeutic setting but that these effects did not generalize to the settings in which probably blinded assessments were made. However, in a substantial minority of trials (Table 1), especially those with an active control arm, probably blinded measures were collected in the treatment setting, and the effects for these trials were no larger than those for trials in which they were collected in a different setting.

The trials included in the meta-analysis used a wide range of training approaches targeting different neuropsychological processes. There was a sufficient number of trials to look at 2 classes of intervention individually, which was not possible in the previous meta-analysis by Sonuga-Barke *et al.*^14^: namely, training of working memory only, and training focusing on multiple neuropsychological domains. The results for trials implementing working memory training only departed in a striking way from the most proximal/probably blinded pattern described above. Effects on ADHD were negligible even considering most proximal measures. This suggests that this form of training, which has been widely promoted for use with patients with ADHD (as discussed by Rapport *et al.*,^9^), has little or no efficacy for core ADHD symptoms. On the other hand, the SMD for most proximal assessment of ADHD symptoms was substantially larger for trials based on training targeting multiple domains than for all studies as a whole. Unfortunately, there was an insufficient number of trials (n = 4) with probably blinded measures to corroborate these effects using independent sources. The superiority of these approaches may be due to the typically greater number of training sessions in multi- compared to single-component approaches (in our analysis, an average of 9 weeks compared to 6 weeks, respectively). However, the finding opens up the interesting possibility that multi-component training models may be more successful for ADHD, given the complex and heterogeneous nature of the condition. Because children with ADHD differ from one another in their neuropsychological profile, and because children may be affected by more than 1 deficit,^37,38^ multi-component training may be used to target a series of neuropsychological domains that may be more important than working memory alone in the pathophysiology of ADHD symptoms. The development and evaluation of multi-component training models should be a future priority.

The effects on neuropsychological outcomes were restricted to working memory, which were substantial, with no effects on inhibitory or attentional control. There were significant effects on parents’ ratings of executive function, but these could not be corroborated by independent blinded evidence. All 6 trials that included a working memory outcome were working memory training trials. Therefore, although these trials produced “near transfer” of training effects to untrained working memory measures, there was no evidence of “far transfer” to other neuropsychological processes. Crucially, there was also no evidence that these effects generalized to important areas of everyday functioning, which themselves are influenced by working memory ability,^16^ such as reading and arithmetic. This finding may be relevant in clinical practice. Indeed, parents may currently favor cognitive training with the hope that they can improve academic performance. Our results show that this is not supported by empirical evidence.

The success of working memory training in improving working memory performance draws into even sharper relief its failure to improve ADHD symptoms, suggesting dissociation between neuropsychological functioning and disorder. There are 4 possible explanations for this: first, that working memory deficits do not, in fact, mediate ADHD pathophysiology^39^; second, that although they do mediate the development of ADHD, they have become entrenched and not susceptible to the type of training implemented in trials conducted to date; third, that training as currently implemented targets types of working memory not fundamental to the deficits in ADHD^9^; and fourth, that training produces only peripheral, practice-like effects on working memory, with no profound impact on the brain networks underpinning neuropsychological deficits responsible for ADHD. Whether or not working memory deficits are part of the causal mechanism underpinning ADHD, based on our results, strengthening working memory appears to be neither a necessary nor a sufficient condition for ADHD symptom reduction. In this regard, our findings suggest that choosing substrates that have emerged from experimental research as treatment targets may not necessarily translate into clinical benefits. This possible dissociation between candidate mechanisms of a disorder and clinical targets is important when adopting pathophysiology-based research approaches such as the Research Domain Criteria (RDoC).^40^ From a clinical standpoint, developing techniques to extend transfer from the effects on core working memory processes to broader neuropsychological processes and important domains of impairment and/or clinical presentation is the most pressing challenge for the future. The reasons for the lack of effect on inhibitory and attentional control are hard to determine on the basis of the current analysis, given the small number of trials that specifically targeted these domains. Although we might predict that training targeting multiple deficit domains would show effects on these neuropsychological processes, there were insufficient trials with multi-component training and measures of inhibition and/or attention to test this. Approaches focusing on motivational or energetic processes may also be valuable (i.e., training to increase delay of gratification).^41^

A number of limitations need to be taken into account when interpreting the current analysis. First, there was significant SMD heterogeneity for some analyses (most proximal total ADHD, symptoms of inattention, and visual working memory). This leaves open the possibility that cognitive training may be effective under specific circumstances in individual trials. Given the limited number of trials available, we were unable to identify specific features of positive trials (apart from therapeutic content, working memory training). Second, only a minority of trials (n = 5) reported using intention-to-treat analyses, a situation that may have inflated the effects for some outcomes, as participants who are more difficult to treat or who perceive the treatment as less beneficial may drop out of trials; however, drop-out was relatively low in most trials. Third, despite the recent substantial increase in the number of available cognitive training trials, there was an insufficient number of trials to evaluate training approaches targeting specific neuropsychological constructs other than working memory training. Fourth, there were insufficient trials to run analyses for some important outcomes (e.g., functional impairment, IQ), as well as for sensitivity analyses or analyses restricted to probably blinded measures for a number of outcomes. Fifth, too few trials included long-term outcomes (Table 1) to allow an evaluation of the extent to which effects on clinical symptoms grew over time or effects on neuropsychological processes persisted. Sixth, no trials were restricted to individuals with both ADHD and the specific neuropsychological deficit to be trained. As a consequence, effect sizes for both neuropsychological deficits and ADHD symptoms may have been truncated: in the former case because there would be little room for improvement where no deficit existed; in the latter case because targeting a neuropsychological deficit that was not causing the condition would be unlikely to reduce symptoms of the core condition. Seventh, in the neuropsychological domains, diverse measures from different tasks (still tapping the same domain, however) were combined across studies to allow the calculation of pooled SMD estimates. Eighth, it is important to understand whether initial symptom-related and neuropsychological treatment effects persist over time and generalize to other domains, if they do. There were insufficient trials that examined long-term outcomes to address this issue. Finally, the categorization of studies as “probably blinded,” although carried out according to previously agreed and clear decision rules set out in the protocol, is limited by an inevitable degree of uncertainty because of limitations in the information reported in some trials.

In summary, the current meta-analysis found limited evidence for the clinical value of cognitive training for children with ADHD outside of the narrow confines of specific targeted neuropsychological processes (i.e., working memory training improved working memory function). Given the evidence for neuropsychological heterogeneity in ADHD, future efforts should be directed at developing protocols to target a broader range of neuropsychological deficits. Furthermore, therapeutic innovation is required to enhance the “far transfer” of specific neuropsychological gains to everyday patterns of functional impairment through more ecologically valid training approaches.^42^ Future trials should more consistently include active control arms, a broader range of functional outcomes, and long-term follow-up.

## Figures and Tables

**Table 1:** Characteristics of Studies Included in the Meta-Analysis

Trial	Design		Training			Sample			Outcomes			
First Author (Year)	Type	Control	Length of Training (Days) and FU	Type	Setting	n T C	Meds T (%) C (%)	Age (mo)	ADHD M-Prox	ADHD P-Blind	Included Neuropsychology Outcomes	Academic Functioning
Klingberg (2005)^26^	2 groups	NA-WMT	35 FU: 3 mo	WMT *RoboMemo*^43^	School/home	26 27	0^a^ 0	116 (mean)	Parent	Teacher	Digit span (verbal WM); span board (visual WM); stroop accuracy (inhibition)	N/A
Shalev (2007)^27^	2 groups	Computer games	56 No FU	Attention training	Clinic	20 16	0^b^ 0^b^	72–156	Parent	Parent	N/A	In-house tests
Johnstone (2010)^28^	2 groups	NA-WMT	35 No FU	Inhibitory and WMT	Home	20 20	47^c^ 78^c^	95–149	Parent	Parent	No go errors % (inhibition)	N/A
Rabiner^d^ (2010)^29^	3 groups	Waitlist	98 FU: within 1 y	Attention training *Captain’s Log*^44^	School/home	25 25^e^	7	NS	Teacher	N/A	N/A	Woodcock-Johnson test
Steiner^f^ (2011)^20^	3 groups	Waitlist	120 No FU	Attention/WMT *BrainTrain*^44^	School	13 15	60	148.8 ± 10.8 (mean)	Parent	Teacher	N/A	N/A
Tucha^g^ (2011)^30^	3 groups	Visual perception training	28 No FU	Attention training *AixTent*	Welfare service, home or lab	16 16	100 100	124–138	N/A	N/A	Vigilance omissions (inattention)	N/A
Johnstone^h^ (2012)^31^	3 groups	Waitlist	35 FU: 6 wk	Adaptive inhibitory training and WMT	Home	22 20	90	95–145	Parent	NA	Counting span (verbal WM); Go NoGo, RT incongruent stimuli (inhibition); oddball task correct (attention)	N/A
Gray (2012)^22^	2 groups	Adaptive math training *Academy of Math*	35 No FU	Adaptive WMT *RoboMemo*^43^	School	32 20	98	144–204	Teacher	N/A	Digit span back (verbal WM); CANTAB spatial WM (visual WM); D2 test total (attention)	Wide-Range Achieve- ment
Green (2012)^32^	2 groups	NA-WMT	25 No FU	Adaptive WMT *RoboMemo*^43^	Home	12 14	67 14	84–168	Parent	Parent	WISC index (verbal WM)	N/A
Van der Oord (2012)^33^	2 groups	Waitlist	35 FU: 9 wk	Adaptive EF training (inhibition, WM, flexibility)	Home	18 22	66	96–144	Parent	Teacher	N/A	N/A
Tamm (2013)^34^	2 groups	Waitlist	56 No FU	Adaptive attention training *Pay Attention!*	School	45 46	65 73	84–180	Parent	Clinician	Digit span (verbal WM); D-KEFS scaled score (inhibition), omissions (inhibition)	N/A
Chacko (2013)^35^	2 groups	NA-WMT	35 No FU	Adaptive WMT *RoboMemo*^43^	Home	44 41	27 32	84–132	Parent	Teacher	AWMA listening (verbal WM); dot matrix (visual WM); CPT commissions (inhibition); omissions (attention)	Wide-Range Achieve-ment
Egeland^i^ (2013)^24^	2 groups	TAU	25 FU: 8 mo	Adaptive WMT *RoboMemo*^43^	School	33 34	68	120–144	Teacher	N/A	Stroop interference score (inhibition; CPT focus (attention)	Logos Test
Hovik (2013)^25^	2 groups	TAU	25 FU: 8 mo	Adaptive WMT *RoboMemo*^43^	School	33 34	68	120–144	Teacher	N/A	Digit span (verbal WM); Leiter visual span (visual WM)	N/A
Steiner^f^ (2014)^21^	3 groups (neurofeed-back, cognitive training, control)	TAU	91 No FU	Adaptive attention and WMT	School	34 36	41 55	100.8 ± 14.8 (mean)	Parent	Direct observa-tion (BOSS)	N/A	N/A
Van Dongen-Boomsma (2014)^36^	2 groups	NA-WMT	35 No FU	Adaptive WMT (Cogmed *RoboMemo*^43^)	Home, except for 1 participant	26 21	0 0	71.5–87.6	Investigator	Teacher	Digit span (verbal WM); Knox Cubes (visual WM); Stroop difference (inhibition); Sustained attention dots: SDRT (attention)	N/A

Note: Studies are listed in chronological order of publication and are followed by study reference number, as in Table S1, available online; long-term follow-up is listed under “Length of Training” after first outcome measurement when available, and “n” is the number of individuals in the treatment (T) and control (C) conditions. ADHD = attention-deficit/hyperactivity disorder; AWMA = automated working memory assessment; BOSS = Behavioral Observation of Students in Schools; CANTAB = Cambridge Neuropsychological Test Automated Battery; D-KEFS = Delis-Kaplan Executive Function System; EF = executive functions; FU = follow-up; MProx = most proximal rater; N/A = not applicable; NA-WMT = non-adaptive working memory training; NS = not specified; PBlind = probably blinded rater; RT = reaction time; SDRT = Spatial Delayed Response Task; TAU = treatment as usual; WISC = Wechsler Intelligence Scale for Children; WMT = working memory training.

a Two children discontinued stimulants more than 1 year before the study, 1 child discontinued stimulant medication 1 week before the study, and the other participants were stimulant naive.

b Four participants in the treatment group and 3 in the control group received psychostimulants throughout the duration of the study. None were medicated either during the training sessions or during the pre- and posttesting sessions.

c Participants were asked to refrain from taking ADHD medication in the 24 hours before testing.

d This study also included an arm on computer-assisted instruction that was not considered for the present meta-analysis.

e A total of 27 additional participants were allocated to computer-assisted instruction.

f This trial also included an arm of neurofeedback (www.playattention.com).

g Results of this study are also reported in Lange KW, Tucha L, Hauser J, Lange KM, Stasik D, Tucha O. Attention training in attention deficit hyperactivity disorder. Aula Abierta 2012;40:55-60.

h This study also included a “software with attention monitoring” arm, which was not included in the present meta-analysis for consistency with interventions included in the other studies retained in the meta-analysis.

i This paper and Hovik *et al*.^14^ refer to the same study and present analyses on different outcomes.

**Table 2:** Summary of Results Showing Pooled Standardized Mean Differences (SMD) Between Treatment and Control Arms for Each Outcome

Outcome	Trials Included	Measure	Study n	Effect			Heterogeneity	
				SMD	95% CI	*p*	I^2^	*p*
ADHD total	All	MProx	14	**0.37**	**0.09 to 0.66**	**.01**	**71**	**<.001**
		PBlind	11	**0.20**	**0.01 to 0.40**	**.04**	30	.16
	Active control	MProx	7	0.16	0.23 to 0.55	.41	**71**	**<.001**
		PBlind	6	0.22	−0.09 to 0.53	.17	42	.13
	WMT	MProx	6	0.00	−0.31 to 0.31	1.00	56	.05
	MPT	MProx	5	**0.79**	**0.46 to 1.12**	**<.001**	36	.18
	MED	MProx	5	0.19	−0.16 to 0.54	.30	56	.06
		PBlind	5	0.11	−0.10 to 0.32	.31	0	.74
Inattention	All	MProx	11	**0.47**	**0.14 to 0.80**	**<.01**	**76**	**<.001**
		PBlind	9	0.32	−0.01 to 0.66	.06	**69**	**<.001**
	Active control	MProx	5	0.30	−0.17 to 0.76	.21	**72**	**<.001**
	WMT	MProx	5	0.22	−0.18 to 0.62	.28	**66**	**<.001**
	MED	MProx	5	0.35	−0.09 to 0.79	.29	**71**	**.02**
Hyper/Imp	All	MProx	9	0.14	−0.07 to 0.35	.18	28	.28
		PBlind	8	0.18	−0.01 to 0.37	.06	0	.50
	Active control	MProx	5	0.01	−0.25 to 0.22	.91	0	.60
	WMT	MProx	5	0.02	−0.24 to 0.21	.89	0	.68
Executive function rating	All	MProx	6	**0.35**	**0.08 to 0.61**	**.01**	22	.22
Working memory (visual)	All	Objective	5	**0.47**	**0.23 to 0.70**	**<.01**	**69**	**<.001**
	Active control	Objective	Insufficient trials (n = 4)					
	WMT	Objective	5	**0.47**	**0.23 to 0.70**	**<.01**	**69**	**<.001**
Working memory (verbal)	All	Objective	8	**0.52**	**0.24 to 0.80**	**<.01**	48	.06
	Active control	Objective	5	**0.58**	**0.23 to 0.94**	**.001**	45	.12
	WMT	Objective	6	**0.57**	**0.29 to 0.82**	**<.001**	32	.19
Inhibition	All	Objective	6	0.07	−0.15 to 0.28	.53	2	.4
Attention	All	Objective	7	0.14	−0.19 to 0.48	.41	**58**	**.03**
Reading	All	Standardized tests	5	0.09	−0.09 to 0.27	.33	23	.26
Arithmetic	All	Standardized tests	5	0.01	−0.13 to 0.11	.84	0	.44

Note: Significant effects are expressed in boldface. Table reports only measures for which 5 or more trials were available. Where only most proximal rater (MProx) is reported, there were insufficient trials with probably blinded rater (PBlind) measures. Active controls = all trials with an active control arm such as easy or non-adaptive training; ADHD = attention-deficit/hyperactivity disorder; All = all trials meeting inclusion criteria with available measure; MED = trials in which <30% of participants were treated with ADHD medication; MPT = multiple process training; WMT = all trials using just working memory training.
