# Erratum

**DOI:** 10.1016/j.jaac.2015.03.010

**Published:** 2015-05

An error appeared in the article “Cognitive Training for Attention-Deficit/Hyperactivity Disorder: Meta-Analysis of Clinical and Neuropsychological Outcomes From Randomized Controlled Trials” by Samuele Cortese, Maite Ferrin, Daniel Brandeis, et al., published in the March 2015 issue of the *Journal of the American Academy of Child and Adolescent Psychiatry* (2015;54:164-174). On page 169, two of the 95% CIs reported in Table 2 contained typographical errors and were inaccurate. The corrected data has been italicized in the table below. The errors do not change the findings of the paper. Nevertheless, to be accurate, the corrected Table 2 appears below in its entirety. The authors and the editorial office regret the error.

**Table 2:** Summary of Results Showing Pooled Standardized Mean Differences (SMD) Between Treatment and Control Arms for Each Outcome

Outcome	Trials Included	Measure	Study n	Effect			Heterogeneity	
SMD	95% CI	*p*	I^2^	*p*
ADHD total	All	MProx	14	**0.37**	**0.09 to 0.66**	**.01**	**71**	**<.001**
		PBlind	11	**0.20**	**0.01 to 0.40**	**.04**	30	.16
	Active control	MProx	7	0.16	*−0.23 to 0.55*	.41	**71**	**<.001**
		PBlind	6	0.22	−0.09 to 0.53	.17	42	.13
	WMT	MProx	6	0.00	−0.31 to 0.31	1.00	56	.05
	MPT	MProx	5	**0.79**	**0.46 to 1.12**	**<.001**	36	.18
	MED	MProx	5	0.19	−0.16 to 0.54	.30	56	.06
		PBlind	5	0.11	−0.10 to 0.32	.31	0	.74
Inattention	All	MProx	11	**0.47**	**0.14 to 0.80**	**<.01**	**76**	**<.001**
		PBlind	9	0.32	−0.01 to 0.66	.06	**69**	**<.001**
	Active control	MProx	5	0.30	−0.17 to 0.76	.21	**72**	**<.001**
	WMT	MProx	5	0.22	−0.18 to 0.62	.28	**66**	**<.001**
	MED	MProx	5	0.35	−0.09 to 0.79	.29	**71**	**.02**
Hyper/Imp	All	MProx	9	0.14	−0.07 to 0.35	.18	28	.28
		PBlind	8	0.18	−0.01 to 0.37	.06	0	.50
	Active control	MProx	5	0.01	−0.25 to 0.22	.91	0	.60
	WMT	MProx	5	0.02	−0.24 to 0.21	.89	0	.68
Executive function rating	All	MProx	6	**0.35**	**0.08 to 0.61**	**.01**	22	.22
Working memory (visual)	All	Objective	5	**0.47**	**0.23 to 0.70**	**<.01**	**69**	**<.001**
	Active control	Objective	Insufficient trials (n = 4)					
	WMT	Objective	5	**0.47**	**0.23 to 0.70**	**<.01**	**69**	**<.001**
Working memory (verbal)	All	Objective	8	**0.52**	**0.24 to 0.80**	**<.01**	48	.06
	Active control	Objective	5	**0.58**	**0.23 to 0.94**	**.001**	45	.12
	WMT	Objective	6	**0.57**	**0.29 to 0.82**	**<.001**	32	.19
Inhibition	All	Objective	6	0.07	−0.15 to 0.28	.53	2	.4
Attention	All	Objective	7	0.14	−0.19 to 0.48	.41	**58**	**.03**
Reading	All	Standardized tests	5	0.09	−0.09 to 0.27	.33	23	.26
Arithmetic	All	Standardized tests	5	0.01	*−0.11 to 0.13*	.84	0	.44

Note: The italicized data in the table indicates information corrected from the original published table. Significant effects are expressed in boldface. Table reports only measures for which 5 or more trials were available. Where only most proximal rater (MProx) is reported, there were insufficient trials with probably blinded rater (PBlind) measures. Active controls = all trials with an active control arm such as easy or non-adaptive training; ADHD = attention-deficit/hyperactivity disorder; All = all trials meeting inclusion criteria with available measure; MED = trials in which <30% of participants were treated with ADHD medication; MPT = multiple process training; WMT = all trials using just working memory training.

