# Supplementary material for: Cognitive Training for Attention-Deficit/Hyperactivity Disorder: Meta-Analysis of Clinical and Neuropsychological Outcomes From Randomized Controlled Trials
Source: J Am Acad Child Adolesc Psychiatry. 2015 Mar;54(3):164–74. doi: 10.1016/j.jaac.2014.12.010 (PMC4382075; doi:10.1016/j.jaac.2014.12.010)
Supplement: Supplementary Data [file mmc1.doc]

**Supplement 1. Search Terms for Each Database**

***PUBMED***

(ADHD OR adhd OR attention deficit disorder with hyperactivity OR minimal brain disorders OR syndrome hyperkinetic OR hyperkinetic syndrome OR hyperactivity disorder OR hyperactive child syndrome OR childhood hyperkinetic syndrome OR attention deficit hyperactivity disorders OR attention deficit hyperactivity disorder OR adhd attention deficit hyperactivity disorder OR addh OR overactive child syndrome OR attention deficit hyperkinetic disorder OR hyperkinetic disorder OR attention deficit disorder hyperactivity OR attention deficit disorders hyperactivity OR child attention deficit disorder OR hyperkinetic syndromes OR syndromes hyperkinetic OR hyperkinetic syndrome childhood) AND (RCT OR cluster RCT OR clinical trial* OR controlled clinical trial* OR crossover procedure OR cross over stud* OR crossover design OR double blind procedure OR double blind method OR double blind stud* OR single blind procedure OR single blind method OR single blind stud* OR random allocation OR randomization OR  random assignment OR randomized controlled trial*) AND (cognitive training OR attention training OR working memory training OR cognitive remediation OR executive function training).

***OVID DATABASES: Medline, PsycINFO, EMBASE+EMBASE CLASSIC***

(ADHD OR adhd OR attention deficit disorder with hyperactivity OR minimal brain disorders OR syndrome hyperkinetic OR hyperkinetic syndrome OR hyperactivity disorder OR hyperactive child syndrome OR childhood hyperkinetic syndrome OR attention deficit hyperactivity disorders OR attention deficit hyperactivity disorder OR adhd attention deficit hyperactivity disorder OR addh OR overactive child syndrome OR attention deficit hyperkinetic disorder OR hyperkinetic disorder OR attention deficit disorder hyperactivity OR attention deficit disorders hyperactivity OR child attention deficit disorder OR hyperkinetic syndromes OR syndromes hyperkinetic OR hyperkinetic syndrome childhood OR Attention deficit disorder / OR ((atteni$) adj3 (deficit$ OR disorder$ or hyperactiv$ OR hyper?activ$ OR adhd OR addh OR ad??hd)) OR ((hyperkin$ OR hyper?kin$) adj3 (deficit$ OR disorder$ OR hkd))) AND (RCT OR cluster RCT OR clinical trial* OR controlled clinical trial* OR crossover procedure OR cross over stud* OR crossover design OR double blind procedure OR double blind method OR double blind stud* OR  single blind procedure OR single blind method OR single blind stud* OR random allocation OR randomization OR  random assignment OR randomized controlled trial*) AND (cognitive training OR attention training OR working memory training OR cognitive remediation OR executive function training).

***ISI WEB of Knowledge***

***(Web of Science [Science Citation Index Expanded], Biological Abstracts, Biosis, Food Science and Technology Abstracts)***

(ADHD OR adhd OR attention deficit disorder with hyperactivity OR minimal brain disorders OR syndrome hyperkinetic OR hyperkinetic syndrome OR hyperactivity disorder OR hyperactive child syndrome OR childhood hyperkinetic syndrome OR attention deficit hyperactivity disorders OR attention deficit hyperactivity disorder OR adhd attention deficit hyperactivity disorder OR addh OR overactive child syndrome OR attention deficit hyperkinetic disorder OR hyperkinetic disorder OR attention deficit disorder hyperactivity OR attention deficit disorders hyperactivity OR child attention deficit disorder OR hyperkinetic syndromes OR syndromes hyperkinetic OR hyperkinetic syndrome childhood) AND (RCT OR cluster RCT OR clinical trial OR controlled clinical trial OR crossover procedure OR cross over study OR crossover design OR double blind procedure OR double blind method OR double blind study OR single blind procedure OR single blind method OR single blind study OR random allocation OR randomization OR random assignment OR randomized controlled trial) AND (cognitive training OR attention training OR working memory training OR cognitive remediation OR executive function training).

***ERIC***

(ADHD OR adhd OR attention deficit disorder with hyperactivity OR minimal brain disorders OR syndrome hyperkinetic OR hyperkinetic syndrome OR hyperactivity disorder OR hyperactive child syndrome OR childhood hyperkinetic syndrome OR attention deficit hyperactivity disorders OR attention deficit hyperactivity disorder OR adhd attention deficit hyperactivity disorder OR addh OR overactive child syndrome OR attention deficit hyperkinetic disorder OR hyperkinetic disorder OR attention deficit disorder hyperactivity OR attention deficit disorders hyperactivity OR child attention deficit disorder OR hyperkinetic syndromes OR syndromes hyperkinetic OR hyperkinetic syndrome childhood) AND (RCT OR cluster RCT OR clinical trial OR controlled clinical trial OR crossover procedure OR cross over study OR crossover design OR double blind procedure OR double blind method OR double blind study OR single blind procedure OR single blind method OR single blind study OR random allocation OR randomization OR  random assignment OR randomized controlled trial) AND (cognitive training OR attention training OR working memory training OR cognitive remediation OR executive function training).

**Supplement 2. Funnel Plots and Egger’s Tests for Meta-Analyses of Effects of Cognitive Training on Attention-Deficit/Hyperactivity Disorder (ADHD) Core Symptoms (Most Proximal and Probably Blinded Measures)**

**Most Proximal Measures**


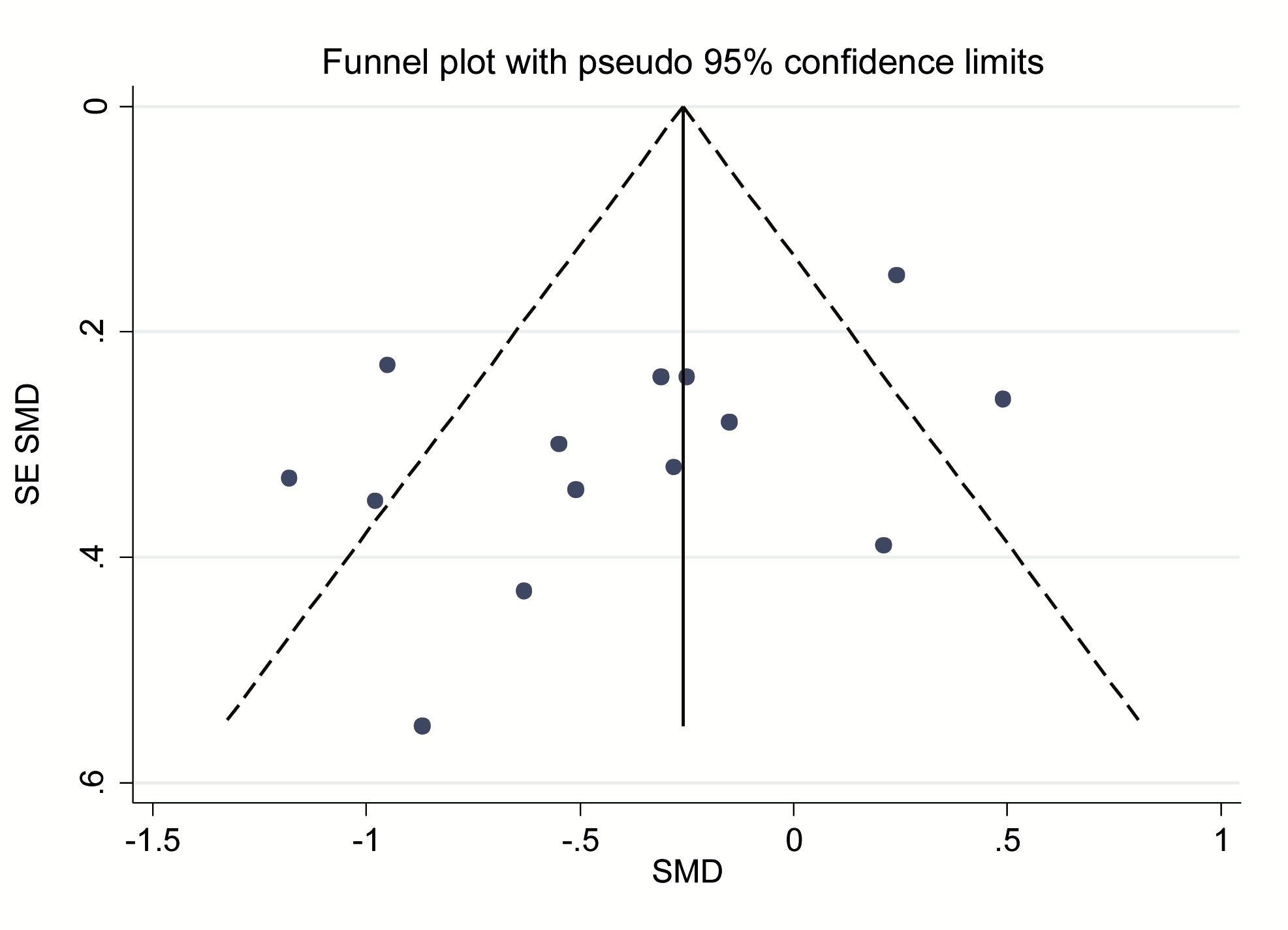


Egger's test for small-study effects:

Regress standard normal deviate of intervention

effect estimate against its standard error

Number of studies = 14 Root MSE = 1.678

----------------------------------------------------------------------------------------------------------

Std_Eff | Coef. SE t *p*>|t| [95% CI]

---------------------------------------------------------------------------------------------------------

slope | .5105885 .3959192 1.29 0.221 -.3520453 1.373222

bias | -2.96456 1.449998 -2.04 0.063 -6.123835 .1947147

---------------------------------------------------------------------------------------------------------

Test of H0: no small-study effects *p* = .063

**Probably Blinded Measures**


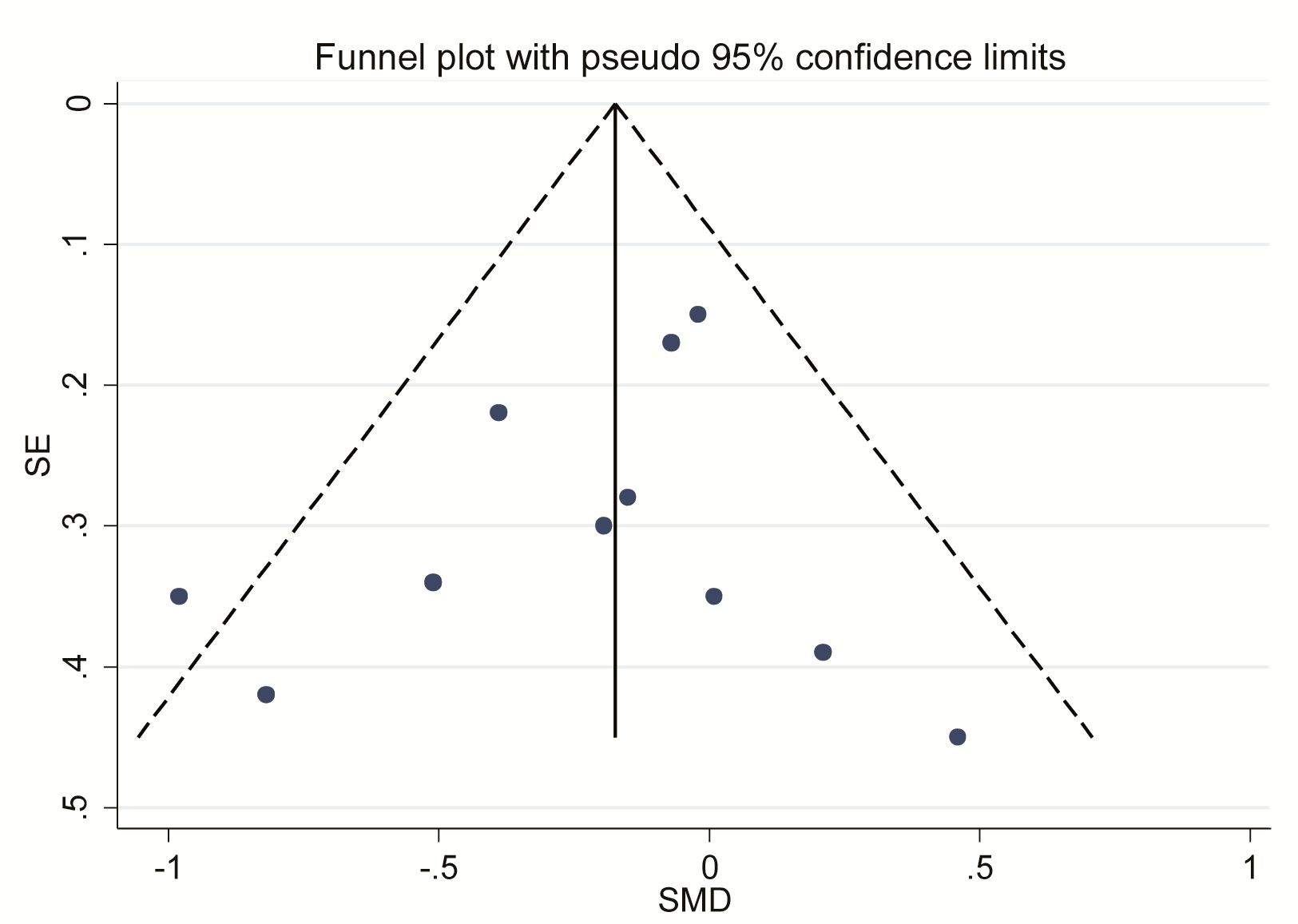


Egger's test for small-study effects:

Regress standard normal deviate of intervention

effect estimate against its standard error (SE)

Number of studies = 11 Root MSE = 1.21

------------------------------------------------------------------------------------------------------

Std_Eff | Coef. SE t *p*>|t| [95% CI]

-------------------------------------------------------------------------------------------------------

slope | .0366061 .2606096 0.14 0.891 -.5529337 .6261459

bias | -.8749447 1.011575 -0.86 0.410 -3.163287 1.413398

-------------------------------------------------------------------------------------------------------

Test of H0: no small-study effects *p* = .410

**Supplement 3. Results of the Meta-Regression Analysis to Assess the Relationship Between Age and Standardized Mean Difference (SMD) for Most Proximal and Probably Blinded Assessment of Attention-Deficit/Hyperactivity Disorder (ADHD) Core Symptoms**

Relationship Between Age and Standardized Mean Difference (SMD) for Most Proximal Attention-Deficit/Hyperactivity Disorder (ADHD) Core Symptoms

| **ID#** | **mean age (months)** | **SMD** | **SE** |
| --- | --- | --- | --- |
| 1 | 116 | -0.55 | 0.3 |
| 2 | 114 | -0.51 | 0.34 |
| 3 | 122 | -0.98 | 0.35 |
| 5 | 148.8 | -0.63 | 0.43 |
| 7 | 120 | -1.18 | 0.33 |
| 8 | 174 | 0.49 | 0.26 |
| 9 | 126 | 0.21 | 0.39 |
| 10 | 132 | -0.87 | 0.55 |
| 11 | 120 | -0.95 | 0.23 |
| 12 | 108 | 0.24 | 0.15 |
| 13 | 132 | -0.25 | 0.24 |
| 15 | 100.8 | -0.31 | 0.24 |
| 16 | 79.5 | -0.28 | 0.32 |

---------------------------------------------------------------------------------------------------------------------

SMD Coef. SE t *p*>|t| [95% CI]

---------------------------------------------------------------------------------------------------------------------

Mean age

(months) 0.0051591 0.0068028 0.76 0.464 -0.0098138; 0.0201319


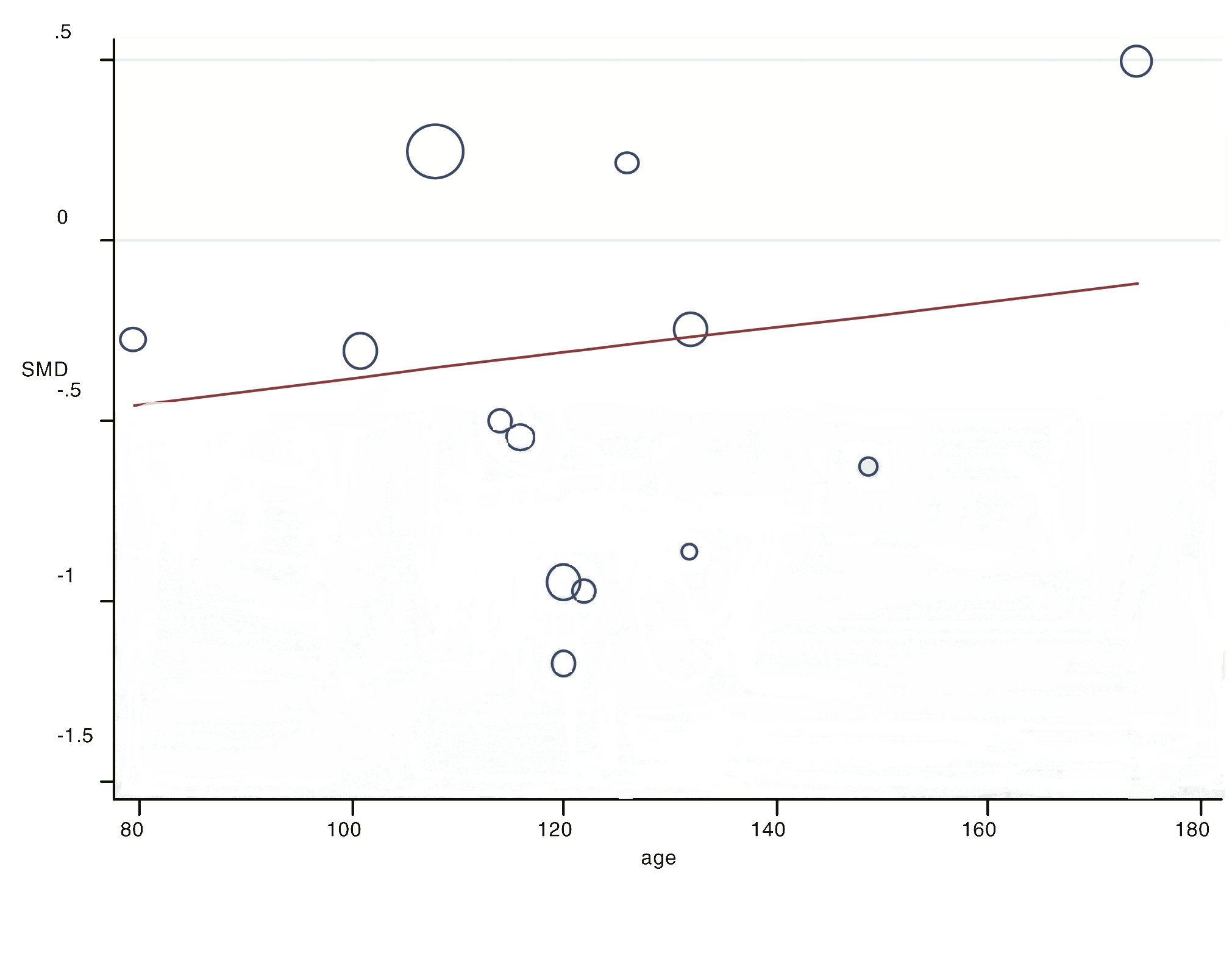


Note: Study ID# refers to ID# reported in Table 1. SE = Standard error.

Relationship Between Age and Standardized Mean Difference (SMD) for Probably Blinded Attention-Deficit/Hyperactivity Disorder (ADHD) Core Symptoms

| **ID#** | **mean age (months)** | **SMD** | **SE** |
| --- | --- | --- | --- |
| 1 | 116 | -0.195 | 0.3 |
| 2 | 114 | -0.51 | 0.34 |
| 3 | 122 | -0.98 | 0.35 |
| 5 | 148.8 | 0.46 | 0.45 |
| 9 | 126 | 0.21 | 0.39 |
| 10 | 132 | -0.82 | 0.42 |
| 11 | 120 | -0.39 | 0.22 |
| 12 | 108 | -0.02 | 0.15 |
| 15 | 100.8 | -0.07 | 0.17 |
| 16 | 79.5 | 0.01 | 0.35 |

-----------------------------------------------------------------------------------------------------------------------

SMD Coef. SE t *p*>|t| [95% CI]

------------------------------------------------------------------------------------------------------------------------

Mean age

(months) -0.004444 0.0081573 -0.54 0.601 -0.0232548 ; 0.0143668


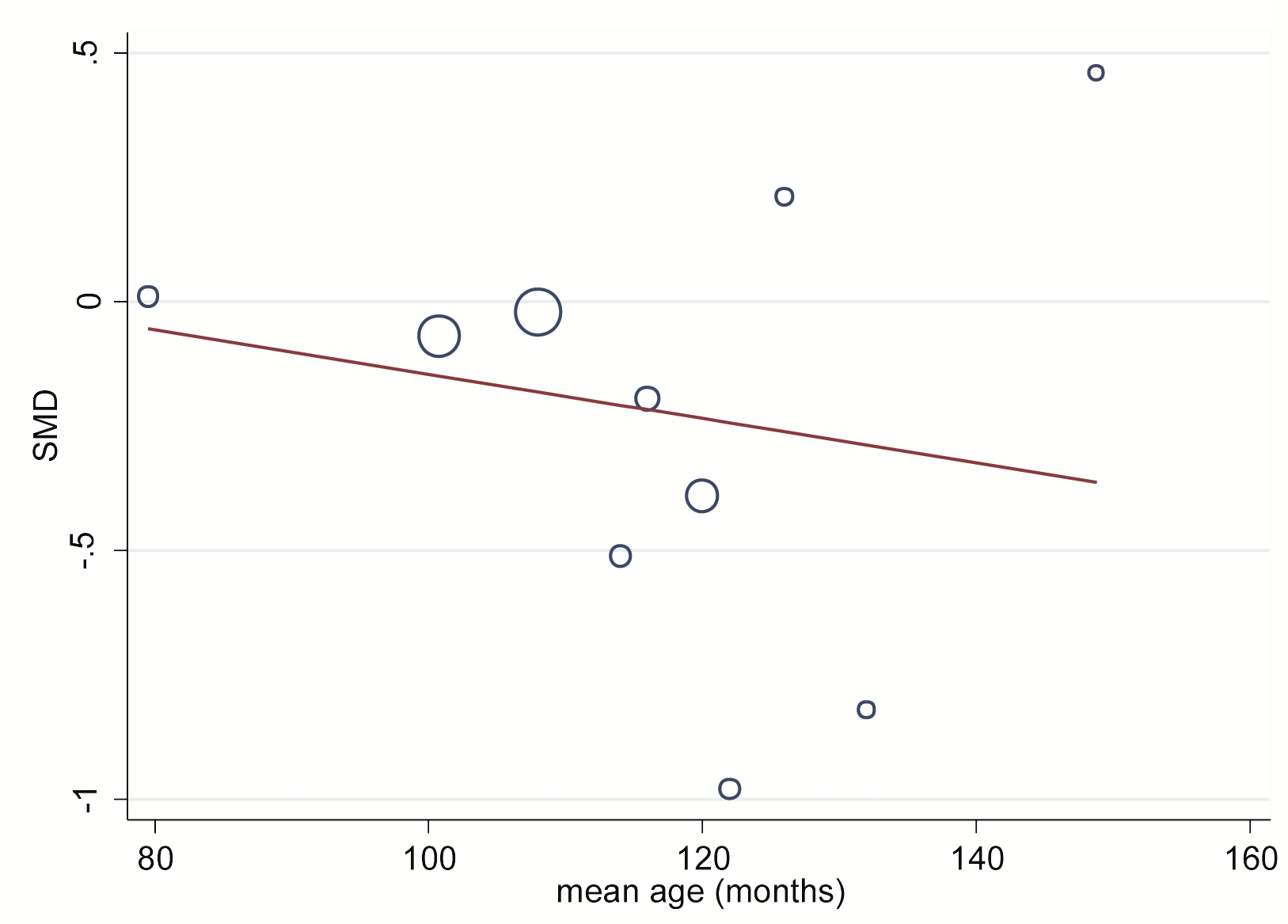


Note: Study ID# refers to ID# reported in Table 1. SE = standard error.

**Table S1. Studies Included in the Meta-Analysis (Listed in Chronological Order of Publication)**

| **Study** | **Reference** |
| --- | --- |
| 1. Klingberg (2005) | Klingberg T, Fernell E, Olesen PJ, et al. Computerized training of working memory in children with ADHD-a randomized, controlled trial. J Am Acad Child Adolesc Psychiatry 2005;44:177-186. |
| 2. Shalev (2007) | Shalev L, Tsal Y, Mevorach C. Computerized progressive Attentional (CPAT) Program: effective direct intervention for children with ADHD. Child Neuropsychology 2006;13:382-388. |
| 3. Johnstone (2010) | Johnstone SJ, Roodenrys S, Phillips E, Watt AJ, Mantz S. A pilot study of combined working memory and inhibition training for children with AD/HD. Atten Defic Hyperact Disord 2010;2:31-42. |
| 4. Rabiner (2010) | Rabiner DL, Murray DW, Skinner AT, Malone PS. A randomized trial of two promising computer-based interventions for students with attention difficulties. J Abnorm Child Psychol 2010;38:131-142. |
| 5. Steiner (2011) | Steiner NJ, Sheldrick RC, Gotthelf D, Perrin EC. Computer-based attention training in the schools for children with attention deficit/hyperactivity disorder: a preliminary trial. Clin Pediatr (Phila) 2011;50:615-622. |
| 6. Tucha (2011) ^a^ | Tucha O, Tucha L, Kaumann G, et al**. Training**of**attention**functions in children with**attention deficit hyperactivity disorder**. **Atten Defic Hyperact Disord** 2011;3:271-8. |
| 7. Johnstone (2012) | Johnstone SJ, Roodenrys S, Blackman R, et al. Neurocognitive training for children with and without AD/HD. Atten Defic Hyperact Disord 2012;4:11-23. |
| 8. Gray (2012) | Gray SA, Chaban P, Martinussen R, et al. Effects of a computerized working memory training program on working memory, attention, and academics in adolescents with severe LD and comorbid ADHD: a randomized controlled trial. J Child Psychol Psychiatry 2012;53:1277-1284. |
| 9. Green (2012) | Green CT, Long DL, Green D, et al. Will working memory training generalize to improve off-task behavior in children with attention-deficit/hyperactivity disorder? Neurotherapeutics 2012;9:639-648. |
| 10. Van der Oord (2012) | Van der Oord S, Ponsioen AJ, Geurts HM, Brink EL, Prins PJ. A Pilot Study of the Efficacy of a Computerized Executive Functioning Remediation Training With Game Elements for Children With ADHD in an Outpatient Setting: Outcome on Parent- and Teacher-Rated Executive Functioning and ADHD Behavior. J Atten Disord 2012, in press, DOI: 10.1177/1087054712453167 |
| 11. Tamm (2013) | Tamm L, Epstein JN, Peugh JL, Nakonezny PA, Hughes CW. Preliminary data suggesting the efficacy of attention training for school-aged children with ADHD. Dev Cogn Neurosci 2013;4:16-28. |
| 12. Chacko (2013) | Chacko A, Bedard AC, Marks DJ, et al. A randomized clinical trial of Cogmed Working Memory Training in school-age children with ADHD: a replication in a diverse sample using a control condition. J Child Psychol Psychiatry 2014;55:247-255. |
| 13. Egeland (2013) | Egeland J, Aarlien AK, Saunes BK. Few effects of far transfer of working memory training in ADHD: a randomized controlled trial. PLoS One 2013;8(10):e75660. |
| 14. Hovik (2013) | Hovik KT, Saunes BK, Aarlien AK, Egeland J. RCT of working memory training in ADHD: long-term near-transfer effects. PLoS One 2013;8:e80561. |
| 15. Steiner (2014) | Steiner NJ, Frenette EC, Rene KM, Brennan RT, Perrin EC. Neurofeedback and cognitive attention training for children with attention-deficit hyperactivity disorder in schools. J Dev Behav Pediatr 2014;35:18-27. |
| 16. Van Dongen-Boomsma (2014) | van Dongen-Boomsma M, Vollebregt MA, Buitelaar JK, Slaats-Willemse D. Working memory training in young children with ADHD: a randomized placebo-controlled trial. J Child Psychol Psychiatry 2014;55:886-96. |

^a^ Results of this study are also reported in Lange KW, Tucha L, Hauser J, Lange KM, Stasik D, Tucha O. Attention training in Attention Deficit Hyperactivity Disorder. Aula Abierta 2012;40(3):55-60.

**Table S2. Studies Excluded From the Meta-Analysis, With Reasons**

| **Study** | **References** | **Reasons for Exclusion** |
| --- | --- | --- |
| Abikoff  (1985) | Abikoff H, Gittelman R. Hyperactive children treated with stimulants. Is cognitive training a useful adjunct? Arch Gen Psychiatry 1985;42:953-961. | No definition of cognitive training according to meta-analysis protocol. |
| Abikoff  (2013) | Abikoff H, Gallagher R, Wells KC, Murray DW, Huang L, Petkova E. Remediating Organizational Functioning in Children With ADHD: Immediate and Long-Term Effects From a Randomized Controlled Trial. J Consult Clin Psychol 2013;81:113-28. | No definition of cognitive training according to meta-analysis protocol. |
| Beck  (2010) | Beck SJ, Hanson CA, Puffenberger SS, Benninger KL, Benninger WB. A controlled trial of working memory training for children and adolescents with ADHD. J Clin Child Adolesc Psychol 2010;39:825-836. | Not randomized. |
| Cho  (2002) | Cho BH, Ku J, Jang D, et al. Clinical test for Attention Enhancement System. Stud Health Technol Inform 2002;85:89-95. | No inclusion criteria for participants. |
| Douglas (1976) | Douglas VI, Parry P, Marton P, Garson C. Assessment of a cognitive training program for hyperactive children. J Abnorm Child Psychol 1976;4:389-410. | No definition of cognitive training according to meta-analysis protocol; not randomized. |
| Gaynor (2013) | Gaynor A, Whitman J, Bessette K, Stevens M. Effects of intensive working memory treatment on brain activity in adolescent combined-subtype ADHD. Biological Psychiatry. Conference: 68th Annual Scientific Convention and Meeting of the Society of Biological Psychiatry, SOBP*.* 2013;73(9 SUPPL. 1). | Not controlled. |
| Gibson  (2011) | Gibson BS, Gondoli DM, Johnson AC, Steeger CM, Dobrzenski BA, Morrissey RA. Component analysis of verbal versus spatial working memory training in adolescents with ADHD: A randomized, controlled trial. Child Neuropsychol 2011;1-18. | Both arms: working memory training. |
| Holmes (2010) | Holmes J, Gathercole S, Place M, Dunning DL, Hilton KA, Elliott JG. Working memory deficits can be overcome: impacts of training and medicaton on working memory in children with ADHD. Appl Cogn Psychol 2009; DOI: 10.1002/acp. | Not controlled. |
| Karatekin (2006) | Karatekin C. Improving antisaccade performance in adolescents with attention-deficit/hyperactivity disorder (ADHD). Exp Brain Res 2006;174:324-341. | No definition of cognitive training according to meta-analysis protocol; not randomized. |
| Kerns  (1999) | Kerns KA, Eso K, Thomson J. Investigation of a direct intervention for improving attention in young children with ADHD. Dev Neuropsychol 1999; 273-295. | Not randomized. |
| Klingberg (2002) | Klingberg T, Forssberg H, Westerberg H. Training of working memory in children with ADHD. J Clin Exp Neuropsychol 2002;24:781-791. | No RCT. |
| Kray  (2011) | Kray J, Karbach J, Haenig S, Freitag C. Can task-switching training enhance executive control functioning in children with attention deficit/-hyperactivity disorder. Front Hum Neurosci 2011;5: 180. | No ADHD outcome. |
| Lloyd  (2010) | Lloyd A, Brett D, Wesnes K. Coherence training in children with attention-deficit hyperactivity disorder: cognitive functions and behavioral changes. Altern Ther Health Med 2010;16:34-42. | No definition of cognitive training according to meta-analysis protocol. |
| Lim  (2012) | Lim CG, Lee TS, Guan C, et al. A Brain-Computer Interface Based Attention Training Program for Treating Attention Deficit Hyperactivity Disorder. PloS One 2012;7:e46692. | Not controlled. |
| Mezzacappa (2010) | Mezzacappa E, Buckner JC. Working memory training for children with attention problems or hyperactivity: a school-based pilot study. School Mental Health 2010; 202-208. | Not controlled. |
| Moore  (1978) | Moore SF, Cole SD. Cognitive self-mediation training with hyperkinetic children. Bull Psychon Soc 1978;18-20 | No definition of cognitive training according to meta-analysis protocol. |
| O'Connell (2006) | O'Connell RG, Bellgrove MA, Dockree PM, Robertson IH. Cognitive remediation in ADHD: effects of periodic non-contingent alerts on sustained attention to response. Neuropsychol Rehabil 2006;16:653-665. | Not randomized. |
| Papazian (2009) | Papazian O, Alfonso I, Luzondo RJ, Araguez N. Training of executive function in preschool children with combined attention deficit hyperactivity disorder: a prospective, controlled and randomized trial. Rev Neurol 2009;48 Suppl 2:S119-S122. | No usable outcome at Time 1. |
| Prins  (2011) | Prins PJ, Dovis S, Ponsioen A, Ten Brink E, Van der Oord S. Does computerized working memory training with game elements enhance motivation and training efficacy in children with ADHD? Cyberpsychology, Behavior, and Social Networking 2011; 14, 115-122. | Both arms: working memory training. |
| Rapport (1996) | Rapport MD, Loo S, Isaacs P, Goya S, Denney C, Scanlan S. Methylphenidate and attentional training. Comparative effects on behavior and neurocognitive performance in twin girls with attention-deficit/hyperactivity disorder. Behav Modif 1996;20:428-430. | Case report. |
| Semrud-Clikeman (1999) | Semrud-Clikeman M, Nielsen KH, Clinton A, Sylvester L, Parle N, Connor RT. An intervention approach for children with teacher- and parent-identified attentional difficulties. J Learn Disabil 1999;32:581-590. | Not randomized. |

Note: ADHD = attention-deficit/hyperactivity disorder; RCT = randomized controlled trial.

**SUPPLEMENTAL FIGURES**

**Figure S1a and b:** Graphic output of the Cochrane risk of bias tool. Note: Detailed ratings of each paper are available upon request.

a.

*
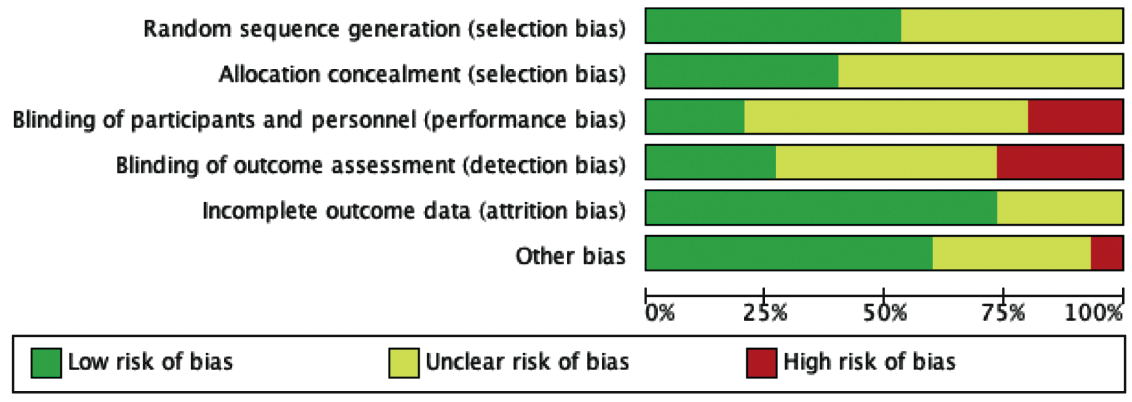
*

b*.*

*
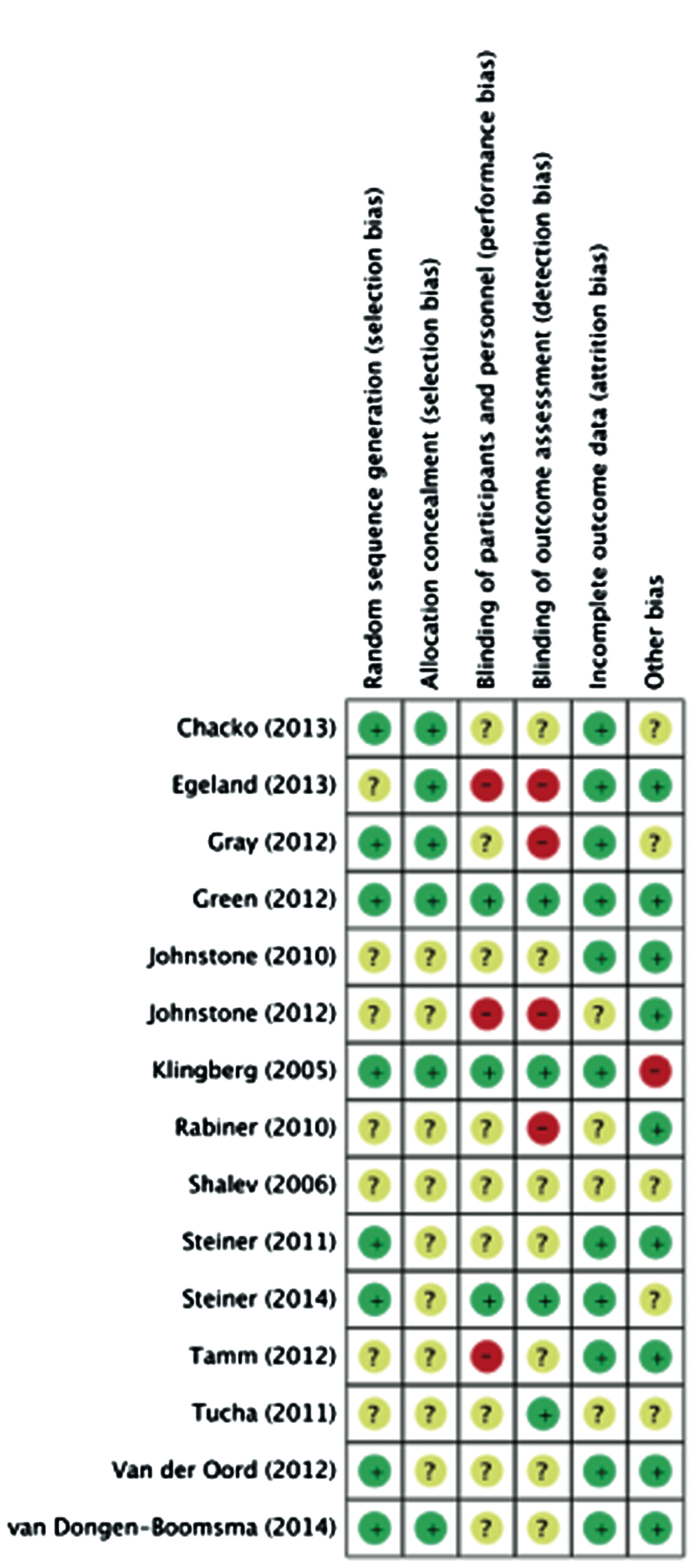
*

**Figure S2:** Forest plots for sensitivity analyses of effects on attention-deficit/hyperactivity disorder (ADHD) core symptoms (total) assessed by most proximal (MPROX) raters. Note**:** References of included studies are listed in Table S1. Std = standard.

*
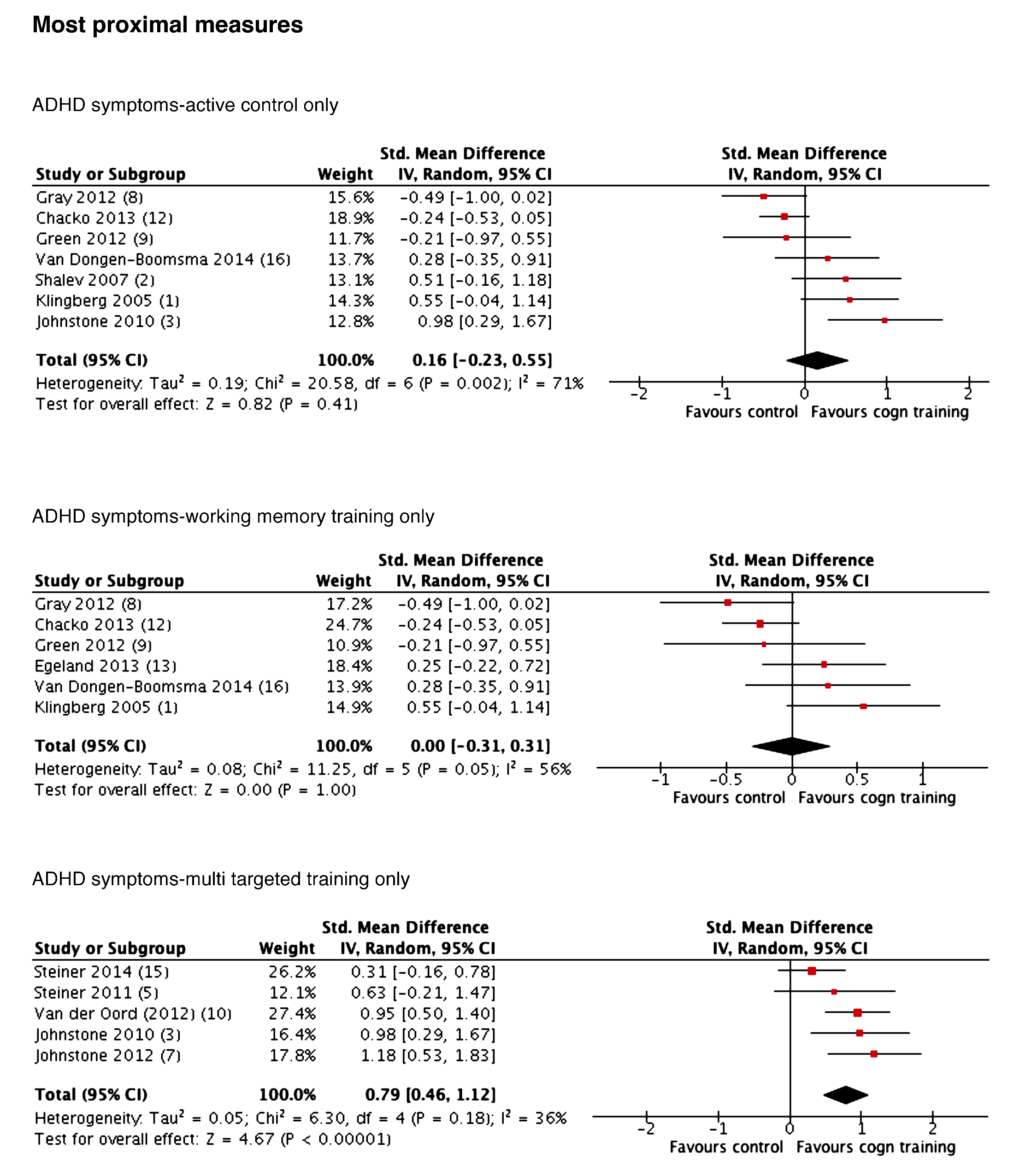
*

**Figure S3:** Forest plots for sensitivity analyses of effects on attention-deficit/hyperactivity disorder (ADHD) core symptoms (inattentive and hyperactive/impulsive) assessed by most proximal (MPROX) raters. Note: References of included studies are listed in Table S1. Cogn = cognitive; Std = standard.

*
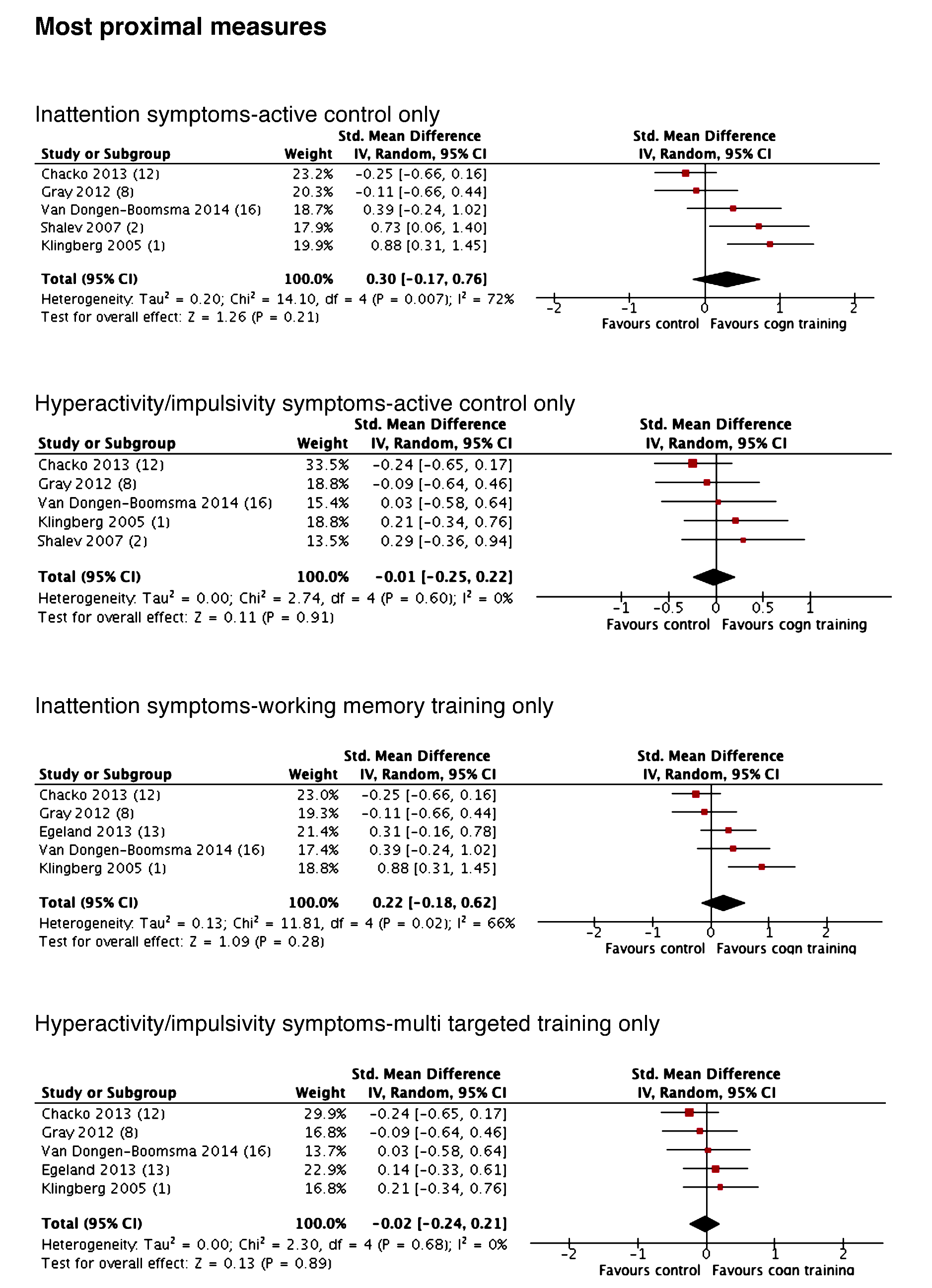
*

**Figure S4.** Forest plot for sensitivity analyses of effects on attention-deficit/hyperactivity disorder (ADHD) core symptoms (total) assessed by probably blinded raters (PBLIND). Note: References of included studies are listed in Table S1. Cogn = cognitive; Std = standard.

*
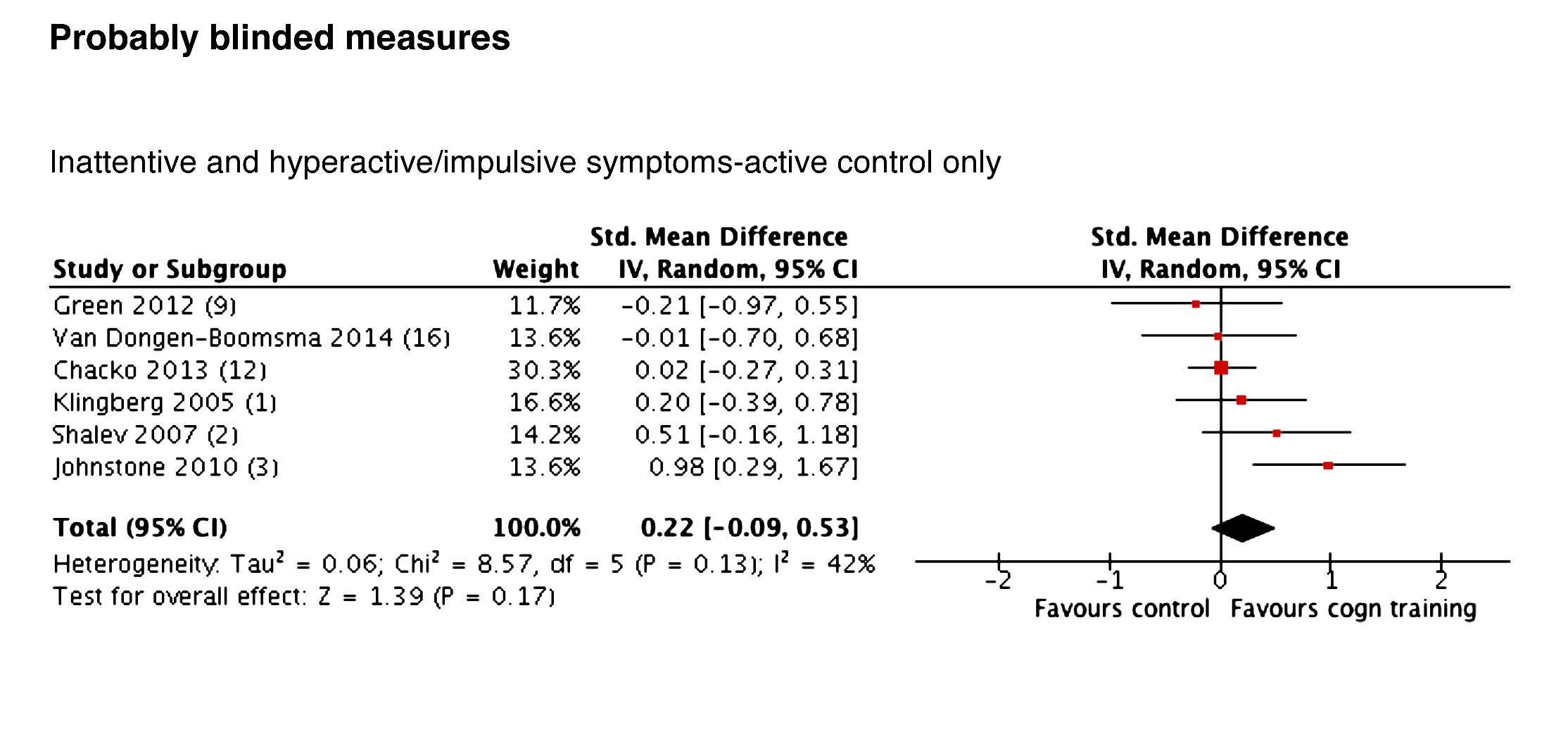
*

**Figure S5.** Forest plots for sensitivity analyses of effects on neuropsychological outcomes. Note: References of included studies are listed in Table S1. Cogn = cognitive; Std = standard.

*
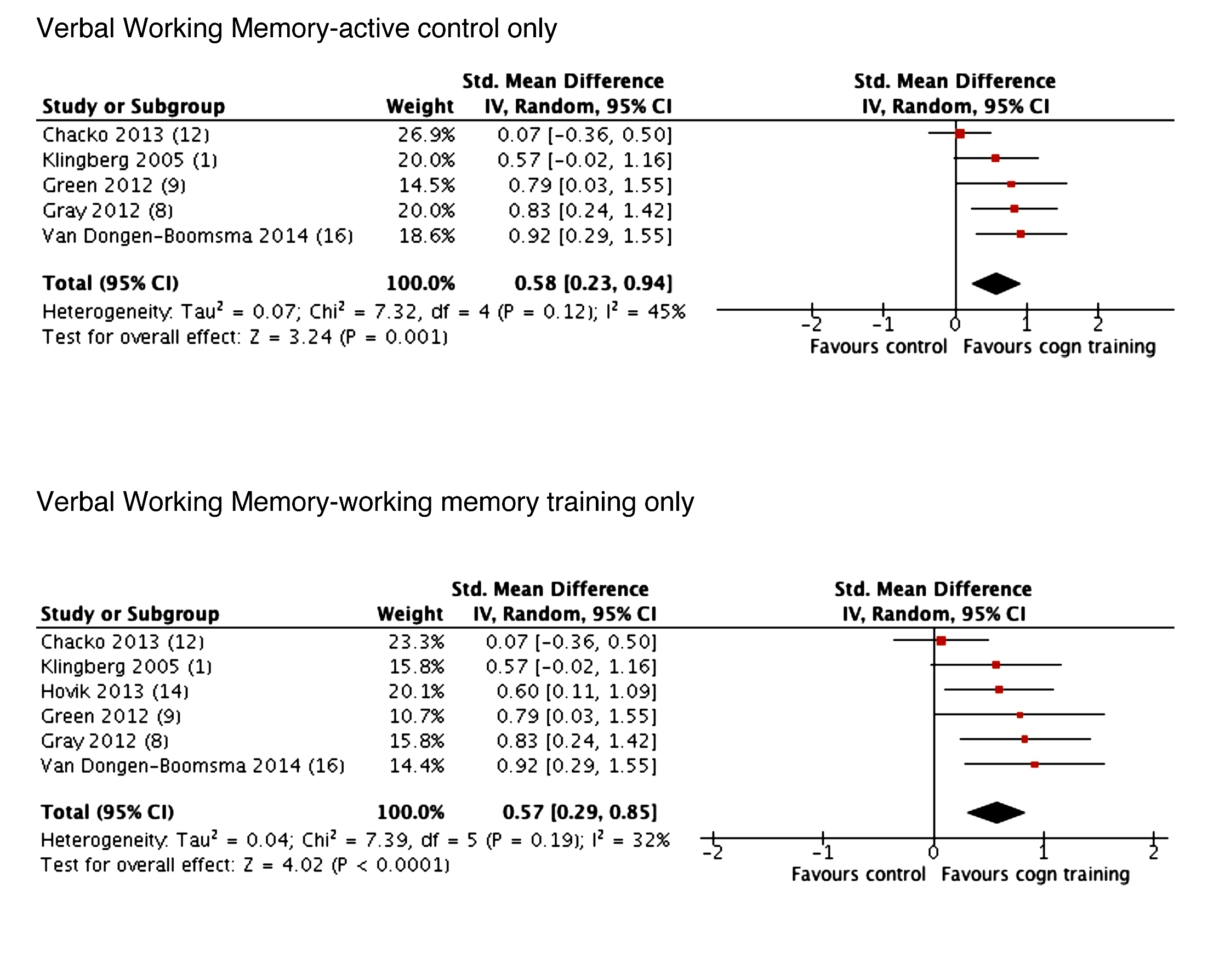
*

**Figure S6.** Forest plot for meta-analysis of effects on the Brief Symptom Inventory, rated by most proximal (MPROX) assessors. Note: References of included studies are listed in Table S1. Cogn = cognitive; Std = standard.

*
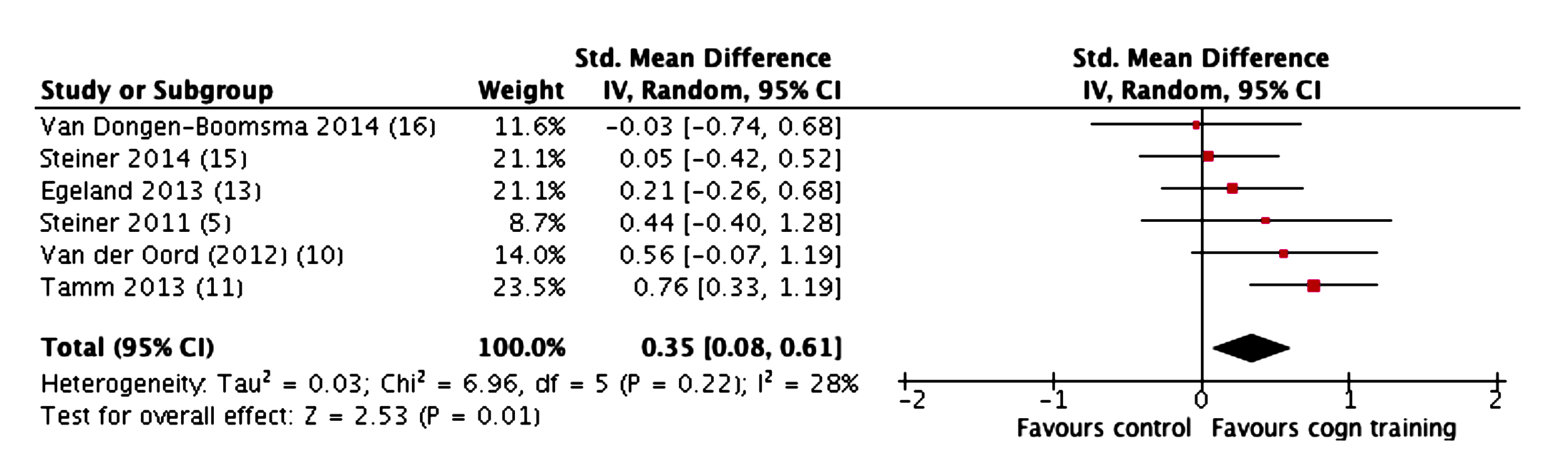
*

**Figure S7.** Forest plot for the sensitivity analysis of the effects of cognitive training on most proximal attention-deficit/hyperactivity disorder (ADHD) core symptoms excluding the study by Gray et al. (see Table S1 for complete reference). Note: Cogn = cognitive; Std = standard.

**
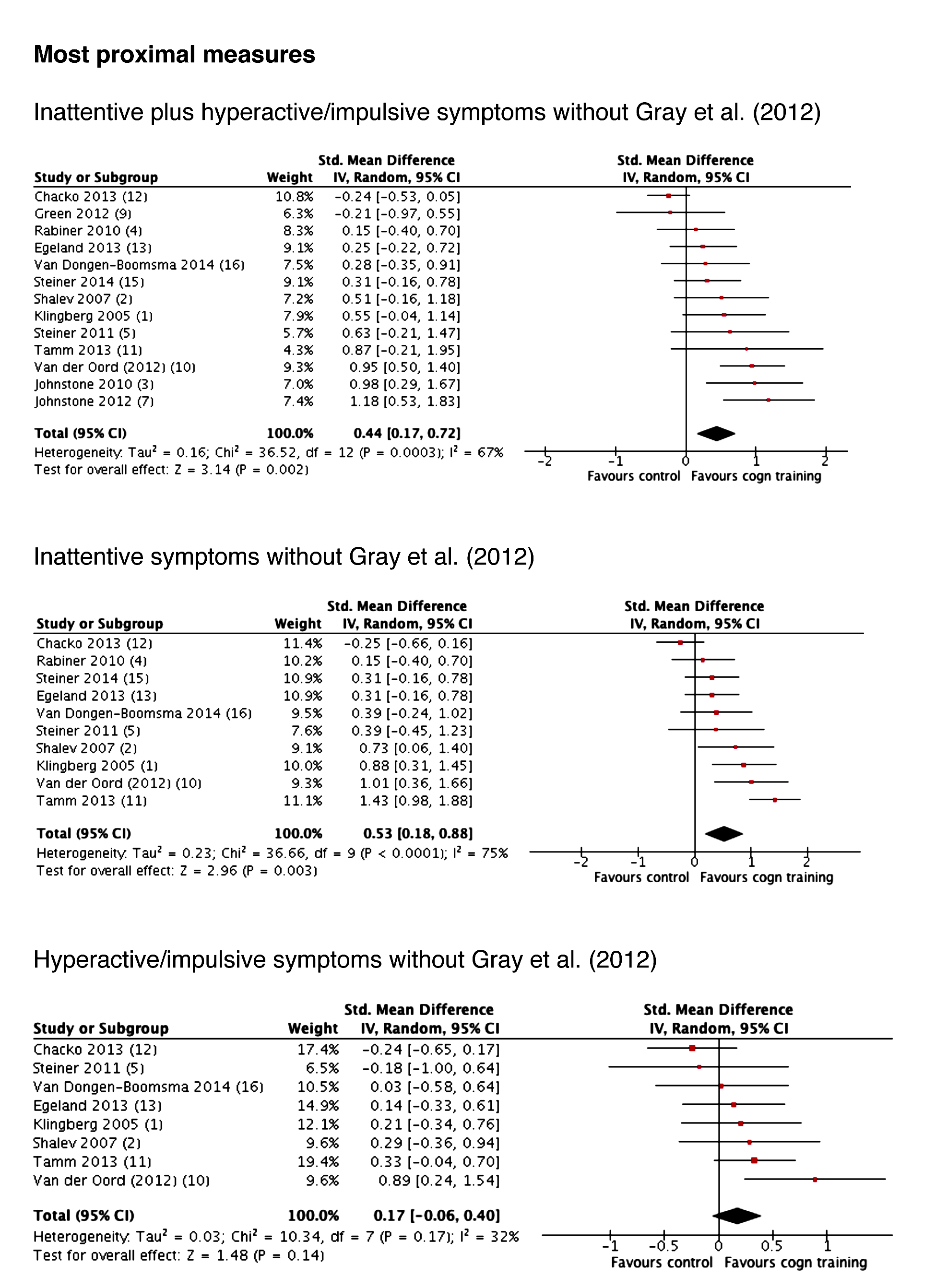
**
